# Unveiling Interleukin-40: A Novel Regulator of Macrophage and B Cell Function in Allergic Asthma

**DOI:** 10.7150/ijbs.128164

**Published:** 2026-06-04

**Authors:** Aixuan Li, Katie Ching-Yau Wong, Danqi Huang, Fang Chen, Haoxuan Li, Xun Gao, Ting-Fan Leung, Gary Wing-Kin Wong, Wing-Hung Ko, Chun-Kwok Wong

**Affiliations:** 1 School of Biomedical Sciences, The Chinese University of Hong Kong, Hong Kong, China.; 2 Department of Chemical Pathology, Prince of Wales Hospital, The Chinese University of Hong Kong, Hong Kong, China.; 3 The Eighth Affiliated Hospital of Sun-Yat-sen University, Shenzhen, China.; 4 Institute of Chinese Medicine, The Chinese University of Hong Kong, Hong Kong, China.; 5 State Key Laboratory of Research on Bioactivities and Clinical Applications of Medicinal Plants, The Chinese University of Hong Kong, Hong Kong, China.; 6 Department of Chemistry, Department of Biochemistry and Molecular Biology, and Institute for Biophysical Dynamics, The University of Chicago, Chicago, IL, USA.; 7 Center of Clinical Laboratory Medicine, Zhongda Hospital, Southeast University, Nanjing, Jiangsu, China.; 8 Department of Laboratory Medicine, Medical School of Southeast University, Nanjing, Jiangsu, China.; 9 Departmenmt of Paediatrics, Prince of Wales Hospital, The Chinese University of Hong Kong, Hong Kong, China.; 10 Li Dak Sum Yip Yio Chin R & D Centre for Chinese Medicine, The Chinese University of Hong Kong SAR, Hong Kong, China

**Keywords:** allergic asthma, airway inflammation, B cell, macrophage, IL-40

## Abstract

Allergic asthma is characterized by chronic airway inflammation and heightened type 2 immune responses. Although inhaled corticosteroids are the mainstay of therapy, a subset of patients exhibits suboptimal responses, underscoring the need for new therapeutic targets. In this study, we investigated the role of novel B cell-related interleukin-40 (IL-40) in allergic asthma using patient samples and a house dust mite (HDM)-induced mouse model. Through transcriptomic and immunological profiling, our findings revealed that IL-40 expression was significantly upregulated in both patients with allergic asthma and murine model. Elevated IL-40 levels exacerbated airway hyperresponsiveness (AHR), promoted inflammatory cell infiltration, and increased the production of type 2 cytokines, indicating a key role in amplifying allergic airway inflammation. Importantly, treatment with a neutralizing antibody against IL-40 or genetic deletion of IL-40 significantly alleviated airway inflammation, suggesting its therapeutic potential. Mechanistically, these pro-inflammatory effects of IL-40 were closely associated with alterations in macrophage polarization and B cell development. Macrophages exhibited the highest induction of IL-40 secretion following allergen exposure and responded most strongly to IL-40 stimulation. This response involved the activation of the JAK/STAT1 and p38-MAPK signaling pathways, driving their polarization toward a pro-inflammatory phenotype while inhibiting the differentiation of a specific Arg1^+^ macrophage subset. Although T cells did not display a direct response to IL-40 stimulation, IL-40 was found to be essential for normal B cell development. IL-40^-/-^ mice showed a marked reduction in pre-B cells in the bone marrow and impaired B cell maturation in the spleen, characterized by decreased follicular B cell populations. Genes involved in B cell receptor synthesis and complement activation were notably downregulated in IL-40^-/-^ mice. These findings position IL-40 as a key regulator of allergic asthma pathogenesis and suggest its potential as a novel biomarker and therapeutic target for airway inflammation.

## Introduction

Asthma is a common chronic airway disease, characterized by airway inflammation, hyperresponsiveness, and symptoms such as wheezing and breathlessness 1. Recent estimates published in The Lancet Respiratory Medicine (2025) reported that there were about 260 million asthma sufferers worldwide in 2021. Furthermore, it is projected that the global number of individuals with asthma will reach 275 million by 2025 2. Allergic asthma, the most prevalent phenotype of asthma, is defined by aberrant immune responses to specific environmental allergens such as house dust mite (HDM), pollen, and pet dander 3, 4. Th2-driven production of immunoglobulin E (IgE) and activation of mast cells and basophils lead to the release of pro-inflammatory mediators, contributing to airway edema, remodeling, and hyperreactivity 5, 6. Therapeutic strategies targeting specific mediators of this Th2-driven response 7, 8, such as dupilumab (blocks IL-4Rα) 9, mepolizumab (neutralizes IL-5) 10, omalizumab (blocks IgE) 11, have demonstrated efficacy in patients with severe and refractory allergic asthma.

In addition to Th2 lymphocytes and eosinophils, B cells and macrophages are increasingly recognized as key contributors to the immunopathogenesis of allergic asthma 12. Airway-resident memory B cells can differentiate into IgE-secreting plasma cells, 13. Disruption of B cell regulatory pathways, such as FoxO1-mediated inhibition of B10 cell differentiation, is linked to enhanced inflammation 14. Macrophages also display functional plasticity and influence airway inflammation and remodeling 15. For instance, factors like histone deacetylase 10 and isthmin-1 modulate macrophage polarization and efferocytosis, shaping inflammatory outcomes 16, 17. Targeting macrophage activity, including through colony-stimulating factor-1 receptor inhibition, offers therapeutic potential. 18. Moreover, our previous work identified IL-41/Metrnl, a macrophage-derived anti-inflammatory cytokine, as a promising therapeutic candidate in allergic asthma 19.

IL-40, a small, secreted protein (~27kDa) encoded by *C17orf99* gene located on chromosome 17, was initially identified as a B cell-associated cytokine mainly expressed in bone marrow and fetal liver. Although the exact function of IL-40 is still unclear, emerging studies suggest that it may participate in both innate and adaptive immune response 20. IL-40 deficient mice display reduced IgA levels in serum, feces, and mammary glands 21. Increased IL-40 expression has been observed in the serum of patients with rheumatoid arthritis (RA) and osteoarthritis, correlating with disease activity and response to therapy 22, 23, and inhibition of IL-40 alleviates sepsis by reducing neutrophil-driven inflammation and multi-organ failure 24. These findings highlight IL-40 as a potential biomarker and immunoregulatory molecule, but its role in allergic asthma remains unexplored.

In this study, we sought to elucidate the regulatory role and underlying mechanisms of IL-40 in allergic asthma using an IL-40 knockout (IL-40^-/-^) mouse model. We further aimed to identify the immune cell populations responsible for IL-40 production and characterize the associated cell types and molecular pathways through a combination of molecular biology techniques and single-cell RNA sequencing. Our findings reveal that IL-40 functions as a pro-inflammatory cytokine in allergic asthma, modulating inflammatory signaling and immune cell dynamics, and may constitute a novel therapeutic target for the management of allergic asthma.

## Materials and Methods

### Patients

We recruited 71 asthmatic children and 34 age- and sex-matched non-allergic healthy controls without any respiratory symptoms or history of allergic asthma at the Department of Paediatrics, Prince of Wales Hospital, Hong Kong (Table S1). Serum specific IgE against 8 common allergens (cockroach, tree mix, cat hair, grass mix, house dust mite Dp1, Alternaria tenuis, mold mix, house dust mite Df1) was measured using the ImmunoCAP-Phadia 100 analyzer (Thermo Fisher Scientific, MA, USA). Children testing positive for specific IgE to any allergen were classified as atopic. The study was approved by the Joint Chinese University of Hong Kong-New Territories East Cluster Clinical Research Ethics Committee (CREC Ref. No.: 2020.641), and conducted in accordance with the Declaration of Helsinki. Written informed consent was obtained from all participants.

### Isolation and stimulation of human peripheral blood mononuclear cell (PBMC)-derived macrophages, B cells and T cells

PBMCs were isolated from fresh human blood buffy coats from healthy volunteers at the Hong Kong Red Cross Blood Transfusion Service by density gradient centrifugation using Ficoll-Paque Plus (Cytiva, MA, USA). PBMCs were seeded in RPMI 1640 medium with 10% FBS and incubated for 24 hours at 37 °C to allow monocyte adhesion. Non-adherent cells were removed by washing with PBS. Adherent monocytes were cultured in RPMI 1640 supplemented with 10% FBS and 50 ng/ml M-CSF for 7 days to generate macrophages, with medium changed every 2-3 days. Macrophages were stimulated with 20 ng/ml IFN-γ and 100 ng/ml LPS for pro-inflammatory M1 phenotype polarization, 20 ng/ml IL-4 and 20 ng/ml IL-13 for anti-inflammatory M2 phenotype polarization, and 200 ng/ml recombinant human IL-40 (rhIL-40) for 24 hours. Unstimulated cells cultured in medium alone served as negative control.

For lymphocyte separation, CD4^+^ T cells were purified from PBMCs using the Human CD4^+^ T cell isolation kit (Miltenyi Biotec, Bergisch Gladbach, Germany). Human B cells were isolated from PBMCs using the EasySep™ Human B Cell Isolation Kit (STEMCELL Technologies, Vancouver, BC, Canada). The purity of isolated T and B cell populations was routinely verified by flow cytometry. Purified T and B cells were stimulated for 24 hours with HDM, CD40L, IL-4 and rhIL-40. Cells and supernatants were collected for further analysis.

Human bronchial epithelial cells (HBEPiC; ScienCell Research Laboratories, Carlsbad, CA, USA) were cultured in Epithelial Cell Medium (ScienCell) supplemented with 10% FBS. After reaching 80% confluence, cells were treated for 24 hours with the indicated stimuli.

### Murine model of experimental allergic asthma

Homozygous C57BL/6J IL-40 knockout mice were generated by Cyagen Biosciences (Santa Clara, CA, USA) and validated by PCR genotyping. Female IL-40^-/-^ and wild-type mice (6-8 weeks old) were maintained under specific pathogen-free conditions at The Chinese University of Hong Kong. Allergic airway inflammation was induced by intranasal sensitization with 1 μg HDM in 30 μl PBS on day 0, followed by daily intranasal challenges with 10 μg HDM in 30 μl PBS from days 8 to 12, with or without 4 μg recombinant mouse IL-40 (MyBiosource, SD, USA). For IL-40 blockade, anti-IL-40 antibody (4 μg) was administered intraperitoneally one day before sensitization and on days 8-12. The antibody was custom-designed and produced by Thermo Fisher Scientific. The antigen used was the IL-40 precursor protein (Mus musculus, Accession #: NP_084240.1, Expression protein project #: 6YJ2660E). The production process included gene synthesis and expression vector creation, small-scale expression optimization, large-scale culture, and tag-based purification. Airway hyperresponsiveness (AHR) was measured on day 13 and mice were anesthetized on day 14. Blood was collected for serum preparation. Bronchoalveolar lavage fluid (BALF) was obtained by flushing the lungs 3 times with PBS (1 ml) via tracheal cannulation. Lung tissues were subsequently harvested for further analysis. All samples were processed or stored appropriately for downstream assays. All animal experiments were conducted following the guidelines outlined by the Animal Experimentation Ethics Committee (AEEC) for the Care and Use of Laboratory Animals and were approved by the AEEC of The Chinese University of Hong Kong.

### AHR monitoring

AHR was assessed using a whole-body plethysmograph (Buxco-Force Pulmonary Maneuvers, Buxco Research Systems, Wilmington, NC, USA). Briefly, unrestrained mice were placed individually into the chambers and exposed to increasing concentrations of aerosolized methacholine (0, 6.25, 12.5, 25, and 50 mg/ml; Sigma-Aldrich) for 3 minutes at each dose. Enhanced pause (Penh) values were recorded for 3 minutes after each challenge to evaluate airway reactivity.

### Histopathology examination

Lung and spleen tissues collected from euthanized mice were fixed in 4% paraformaldehyde, and embedded in paraffin. Sections (5 μm) were prepared for hematoxylin-eosin and PAS staining using an H&E staining kit (Beyotime, Inc., Jiangsu, China) or PAS staining kit (Sigma-Aldrich Corp., Saint Louis, MO, USA), respectively.

### Murine macrophage

Bone marrow cells were harvested by flushing femurs and tibias of euthanized C57BL/6J wild-type and IL-40^-/-^ mice with cold Dulbecco's Phosphate Buffered Saline (DPBS). The cell suspension was filtered through a 70 μm cell strainer and centrifuged at 1,500 rpm for 5 minutes. After red blood cell lysis, cells were cultured in RPMI 1640 medium supplemented with 10% FBS, 1% penicillin-streptomycin, and 20 ng/ml recombinant mouse M-CSF (PeproTech, Cranbury, NJ, USA) at 37°C and 5% CO_2_. The medium was replaced every 2-3 days. After 7 days of culture, adherent bone marrow derived macrophages (BMDM) were gently washed and stimulated for 24 hours with either 100 ng/ml LPS and 20 ng/ml IFN-γ to induce pro-inflammatory M1 polarization, or 20 ng/ml IL-4 and 20 ng/ml IL-13 to induce anti-inflammatory M2 phenotype. An additional group was treated with 200 ng/ml recombinant mouse IL-40 (rmIL-40). Unstimulated cells cultured in medium alone served as negative control.

RAW 264.7 murine macrophage (ATCC, Manassas, VA, USA) cell line was cultured in DMEM containing 10% FBS and 1% penicillin-streptomycin at 37 °C and 5% CO_2_. After seeding and overnight adhesion, cells were treated for 24 hours with the same stimuli as BMDM. Cells and supernatants from all experiments were collected for further analysis.

### Immunofluorescence staining

BMDMs were seeded onto coverslips and fixed with 4% paraformaldehyde. Cells were blocked and incubated with primary antibodies against F4/80 and IL-40 overnight at 4 °C. After washing, samples were incubated with secondary antibodies: Alexa Fluor 488-conjugated anti-F4/80 (green) and Alexa Fluor 647-conjugated anti-IL-40 (red). Nuclei were counterstained with DAPI (blue).

### Isolation of mouse splenic B cells and thymic T cells

Mouse spleens were mechanically dissociated through a 70 μm cell strainer into cold DPBS to obtain single-cell suspensions. After red blood cell lysis, B cells were isolated using the EasySep™ Mouse B Cell Isolation Kit (STEMCELL Technologies). For T cell isolation, thymuses were gently minced and pressed through a 70 μm cell strainer in cold DPBS to generate single-cell suspensions. The purity of isolated B and T cells was routinely confirmed by flow cytometry. Purified T and B cells were stimulated for 24 hours with HDM, CD40L, IL-4 and rmIL-40. Cells and supernatants were collected for further analysis.

### Flow cytometric analysis

To prepare single-cell suspensions, spleens and bone marrow were mechanically dissociated through a 70 μm cell strainer (Corning, NY, USA), and thymus, lymph nodes, and lung tissues were rinsed with PBS, cut into small pieces, and digested in RPMI 1640 containing 5% FBS, 1 mg/ml Collagenase D, and 30 μg/ml DNase I (Roche, Mannheim, Germany) for 15-30 min at 37 °C, and filtrated through a 70 μm cell strainer. Suspensions were subjected to red blood cell lysis, washed, and centrifuged to obtain single cells. Cell pellets were incubated with Fc receptor blocking antibody and stained with fluorochrome-conjugated monoclonal antibodies targeting surface markers (Biolegend Inc. San Diego, CA). For intracellular cytokine detection, cells were fixed, permeabilized, and stained with relevant antibodies. Flow cytometric analysis was performed after sequential gating on singlets, live cells (7-AAD^-^), and CD45^+^ leukocytes. B cells were identified as CD19+B220+, with further delineation using markers including CD21, CD23, CD93, IgM, and IgD to define developmental subsets. B cell activation was also assessed by quantifying CD86^+^ B cells in both mouse and human samples (CD19^+^). Neutrophils were Ly-6G^+^CD11b^+^, eosinophils were SiglecF^+^CD11c^-^, monocytes/macrophages and their polarization subsets were characterized by CD11b, CD11c, F4/80, Arg1, and CD206 markers. Gating for eosinophils in BALF followed singlets, live cells, CD45^+^, CD11b^+^, Ly-6G^-^ and SiglecF^+^. T helper (Th) cell subsets were identified as CD45^+^CD3^+^CD4^+^ cells, with intracellular staining for Th2 (IL-4^+^) and Th17 (IL-17A^+^) populations. Analysis was performed using a FACSVia (BD Biosciences) or Cytek Northern Lights flow cytometer (Cytek Biosciences, CA, USA).

### Quantitative real-time RT-PCR (qRT-PCR)

Total RNA was isolated from tissues or cultured cells using QIAzol reagent (Qiagen Inc., Valencia, CA, USA) and reverse-transcribed into complementary DNA (cDNA) using PrimeScript™ RT Master Mix (Takara Bio Inc., Shiga, Japan). Quantitative real-time PCR was performed using SYBR® Premix Ex Taq™ (Takara Bio Inc.) with GAPDH/ACTIN as the internal control. Relative gene expression levels were calculated using the ΔΔCt method. Primer sequences are provided in Table S2.

### Western blotting

Proteins were extracted from tissues and cells using RIPA lysis buffer (Beyotime, Shanghai, China) containing protease and phosphatase inhibitors. Protein concentrations were determined using a BCA protein assay kit (Thermo Fisher Scientific, Waltham, MA, USA). Equal amounts of protein were separated by SDS-PAGE and transferred onto PVDF membranes (Millipore, Billerica, MA, USA). Protein bands were visualized using an ECL detection kit (Thermo Fisher Scientific) and quantified using ImageJ software. β-Tubulin was used as the loading control.

### Enzyme-linked immunosorbent assay (ELISA)

Concentrations of murine total IgE, anti-HDM IgE, and cytokines including IL-4, IL-5, IL-13, and IL-10 were measured by using ELISA kits (BioLegend). Human and murine IL-40 levels were determined by using ELISA kits (Biorbyt, Cambridge, UK and Shanghai Jianglai Biotechnology Co., Ltd., Shanghai, China). All assays were conducted according to the manufacturers' instructions and quantified based on standard curves.

### Bulk RNA sequencing (RNA-seq) analysis

Total RNA was isolated from murine lung tissue using QIAzol reagent (Qiagen, Inc., Valencia, CA, USA). RNA samples were sent to LC-Biotechnologies Ltd (Hangzhou, China) for library preparation and transcriptomic sequencing. Sequencing libraries were prepared with the NEBNext^®^ Ultra™ RNA Library Prep Kit for Illumina^®^ (Illumina Corp., San Diego, CA, USA) and sequenced on an Illumina HiSeq 4000 platform.

### Single-cell RNA sequencing (scRNA-seq) analysis

Fresh tissues were immediately processed into single-cell suspensions. After rinsing with DPBS (calcium- and magnesium-free), tissues were minced and digested with collagenase D (1 mg/ml) and DNase I (0.2 mg/ml) at 37 °C for 15-25 minutes. The mixture was filtered through a 40 μm strainer, centrifuged, and treated with RBC lysis buffer. Cell viability and concentration were assessed using acridine orange/propidium iodide staining on a Cellometer Auto 2000 and confirmed by trypan blue (> 85% viability). Single-cell libraries were prepared using the Chromium Next GEM Single Cell 3ʹ Reagent Kits v3.1 (10× Genomics), with cells loaded at 1000 cells/μl onto a 10x Genomics Chromium Controller. Sequencing reads were aligned to the mouse genome (mm10) with Cell Ranger (v7.1.0) for barcode identification and quantification. From the lung and bone marrow samples, 14,502 and 14,866 cells, respectively, were obtained before quality control. Each sample generated 330-380 million reads, achieving an average of 40,000-65,000 reads per cell and a median of 828-1,328 genes detected per cell. Over 84% of sequencing reads were assigned to cells, and RNA Q30 base percentages exceeded 94%, indicating high accuracy.

Cells with fewer than 200 or more than 6,000 detected genes were excluded. Only cells with mitochondrial gene content less than 20% (lung) or 5% (bone marrow) were retained, resulting in 13,041 (lung) and 12,816 (bone marrow) high-quality cells for downstream analysis. Data were normalized and scaled, with the top 2000 highly variable genes selected. Principal component analysis (PCA) was conducted on these genes, and batch effects were corrected using the RunHarmony function. Clustering used a k-nearest neighbor graph (k = 20) and the Louvain algorithm (resolution = 0.4), conducted via Seurat (v4). Clusters were visualized with UMAP (first 20 principal components). Cell types were annotated using canonical markers. For dataset integration, Seurat's integration workflow was employed, including further normalization, scaling, and clustering. Comparisons of cell type proportions between groups were visualized using bar plots. Differential gene expression heatmaps, violin plots, cluster heatmaps, and other figures were generated using the https://www.bioinformatics.com.cn online platform. Pseudotime trajectory analysis was performed for B lineage cells in bone marrow using the Slingshot package (v2.12.0), and trajectories were visualized on UMAP plots as smooth lineage curves.

### Statistics

All experiments were conducted with at least three independent biological replicates. Data are presented as mean ± SEM unless otherwise specified. Statistical comparisons between two groups were performed using either a two-tailed Student's t-test or the nonparametric Mann-Whitney U test, depending on data distribution. For multiple group comparisons, one-way ANOVA was used, followed by Tukey's post hoc test. Statistical analyses were carried out using GraphPad Prism 9 (GraphPad Software, San Diego, CA, USA). Statistical significance was defined as P < 0.05.

## Results

### IL-40 expression is up-regulated in allergic asthma

Allergic asthma involves elevated type 2 inflammation and various immune mediators. Previous studies have reported increased serum IL-40 in patients with autoimmune diseases such as RA 22, 25, but its relevance in asthma remained unknown.

**Figure 1:**
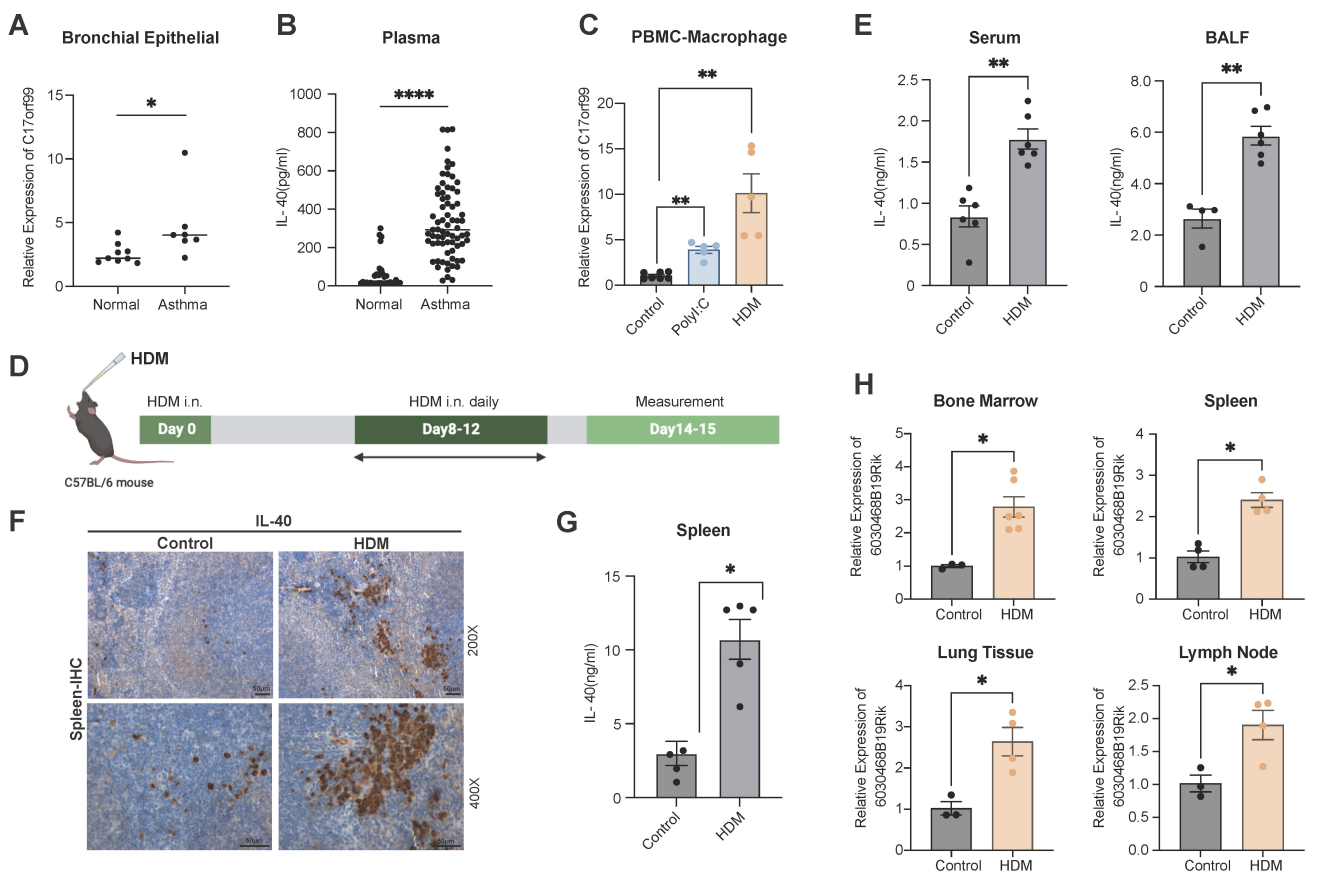
** IL-40 is upregulated in allergic asthma. A** Expression level of *C17orf99* mRNA in human bronchial epithelial cell samples from asthmatic patients (n=7) and healthy controls (n=9), based on GEO dataset (GSE104472). **B** IL-40 concentration in human plasma (asthma patients n=71, healthy controls n=34) measured by ELISA. **C** Expression level of *C17orf99* mRNA in human PBMC-derived macrophages (n=5-7) measured by qRT-PCR. **D** Timeline diagram of the HDM-induced allergic asthma mouse model. **E** IL-40 concentration in serum and BALF from HDM-treated and control mice (n=4-6) measured by ELISA. **F** Representative H&E-stained mouse spleen sections from HDM-treated and control mice. **G** IL-40 concentration in spleen homogenates from mice (n=4-5) measured by ELISA. **H** Expression level of *6030468B19Rik* mRNA in mouse tissues (n=4-6) measured by qRT-PCR. Data are representative of at least three individual experiments. Values are expressed as the mean ± SEM. *P < 0.05; **P < 0.01, and ****P < 0.0001 by Mann-Whitney test.

To clarify IL-40 expression in allergic asthma, we analyzed GEO data (GSE104472) and found significantly increased *C17orf99* (IL-40) mRNA in bronchial epithelial cell samples from asthmatic patients compared to healthy controls (Fig. 1A). Plasma IL-40 was also notably elevated in children with allergic asthma (n = 71) versus healthy donors (n = 34) (Fig. 1B). Among human PBMC-derived immune cells, both B cells and macrophages secreted IL-40 after stimulation, with macrophages showing the strongest response to HDM, while T and epithelial cells contribute minimally (Supplementary Fig. 1A, Fig. 1C), suggesting they could be the primary responding cell type under this allergen exposure.

We further established a murine model of HDM-induced allergic asthma (Fig. 1D, Supplementary Fig. 1B, Supplementary Fig. 1C), IL-40 levels were markedly increased in both serum and BALF of asthmatic mice (Fig. 1E). Given that IL-40 was initially identified as a B cell-associated cytokine 21, we selected the spleen for IHC staining analysis. IHC detected enhanced IL-40 expression in the HDM-treated mice (Fig. 1F). This finding was verified by ELISA (Fig. 1G). Furthermore, the expression of the murine *C17orf99* ortholog *6030468B19Rik* was also elevated in bone marrow, spleen, lung, and lymph node in asthmatic mice (Fig. 1H). IL-40 expression is therefore upregulated broadly in both systemic and local tissues during allergic airway inflammation.

### The pro-inflammatory function of IL-40 in allergic asthma

**Figure 2:**
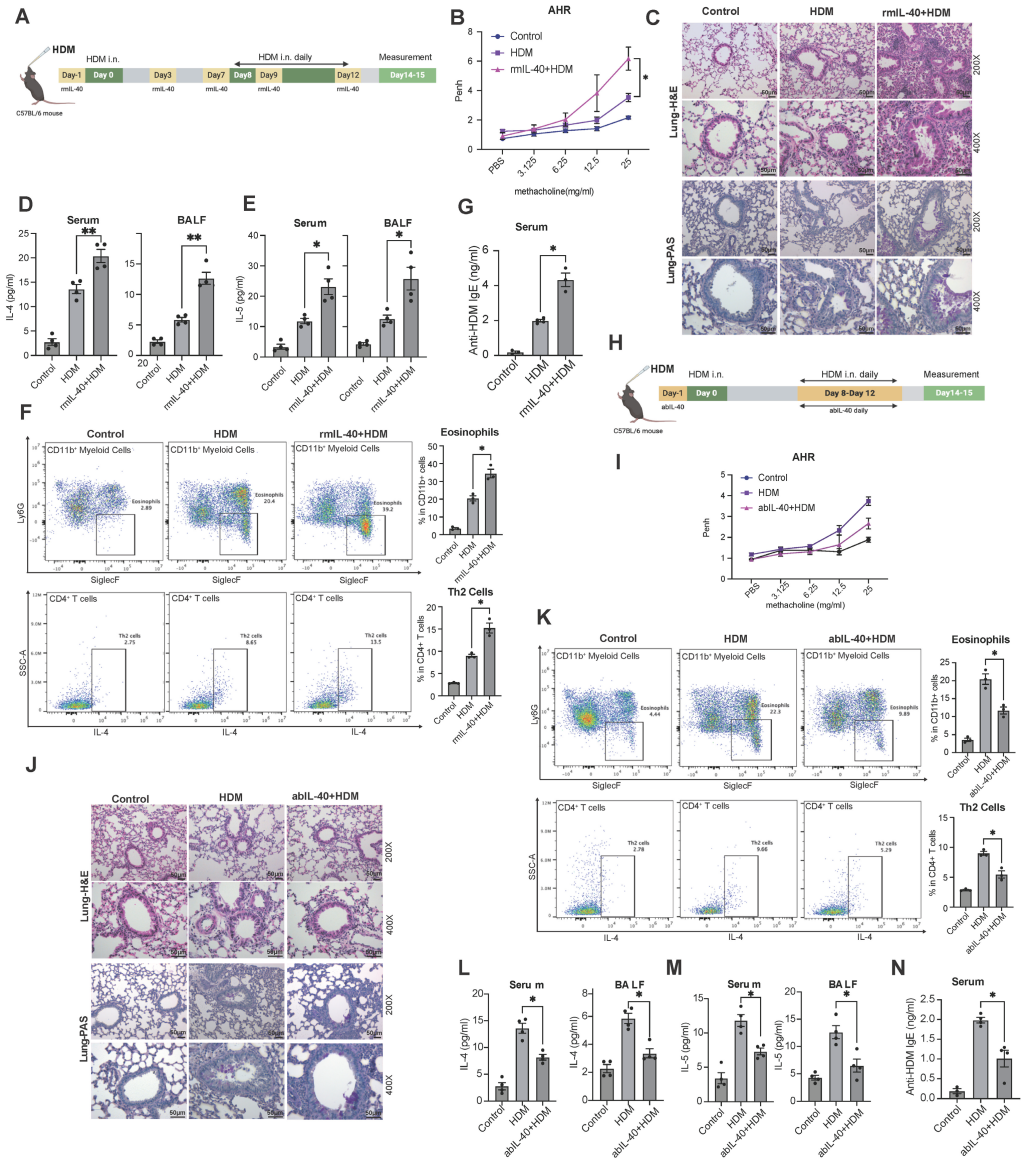
** IL-40 regulates the progression of allergic asthma in a mouse model. A** Timeline diagram of the HDM-induced allergic asthma mouse model with rmIL-40. **B** AHR to methacholine challenge in control, HDM, and rmIL-40+HDM groups (n=4-6). **C** Representative H&E- and PAS-stained lung sections. **D, E** Concentrations of IL-4 and IL-5 in serum and BALF (n=4-6) measured by ELISA, respectively. **F** (rmIL-40),** K** (abIL-40) Eosinophils defined as Ly-6G^-^ SiglecF⁺ cells within the CD11b⁺ population in BALF, and Th2 cells identified as IL-4^+^ cells within the CD45^+^CD3^+^CD4^+^ population in lung tissue; representative flow cytometry plots and quantification of cell proportions are shown (n=3).** G** Concentration of anti-HDM IgE in mouse serum (n=3-5) measured by ELISA. **H** Timeline diagram of HDM-induced asthma mouse model with abIL-40. **I** AHR to methacholine challenge in control, HDM, and abIL-40 + HDM groups (n=4-6). **J** Representative H&E- and PAS-stained lung sections. **L, M** Concentrations of IL-4 and IL-5 in serum and BALF (n=4-6) measured by ELISA, respectively. **N** Concentration of anti-HDM IgE in mouse serum (n=3-5) measured by ELISA. Data are representative of at least three individual experiments. Values are expressed as the mean ± SEM. *P < 0.05; **P < 0.01 by Mann-Whitney test.

The elevated IL-40 expression in both allergic asthma patients and HDM-induced mouse model may reflect a compensatory mechanism aimed at resolving inflammation, or play a pro-inflammatory role in the pathogenesis of allergic asthma 26. To address the precise function of IL-40, we administered rmIL-40 intravenously during HDM-induced asthma modeling in mice (Fig. 2A). Mice treated with rmIL-40 showed aggravated AHR to methacholine, compared to controls (Fig. 2B), and displayed more pronounced airway inflammation and mucus production in lung tissue (Fig. 2C). Serum and BALF levels of type 2 cytokines IL-4 and IL-5 were markedly increased upon rmIL-40 administration (Fig. 2D, E). Flow cytometry further revealed a higher proportion of eosinophils in BALF and increased Th2 cells in lung tissue (Fig. 2F), consistent with enhanced allergic inflammation. Serum anti-HDM IgE was also elevated (Fig. 2G). These findings support the pro-inflammatory role of IL-40 in the allergic asthma model, particularly in driving type 2 immune responses and eosinophilic inflammation.

To eliminate potential confounding effects of exogenous protein administration and further clarify the role of IL-40, we used a neutralizing antibody (abIL-40) in the HDM asthma model (Fig. 2H). IL-40 blockage resulted in reduced AHR (Fig. 2I) and alleviated lung inflammation (Fig. 2J), accompanied by a decrease in tissue eosinophils, Th2 cells, and levels of IL-4, IL-5, and anti-HDM IgE (Fig. 2K-N).

Taken together, these results demonstrate that IL-40 promotes allergic airway inflammation by enhancing Th2 cytokine secretion, eosinophil recruitment, and antigen specific IgE production, confirming its pro-inflammatory role in allergic asthma.

### IL-40^-/-^ mice were less susceptible to develop allergic asthma-associated inflammatory phenotypes following HDM-induction

**Figure 3:**
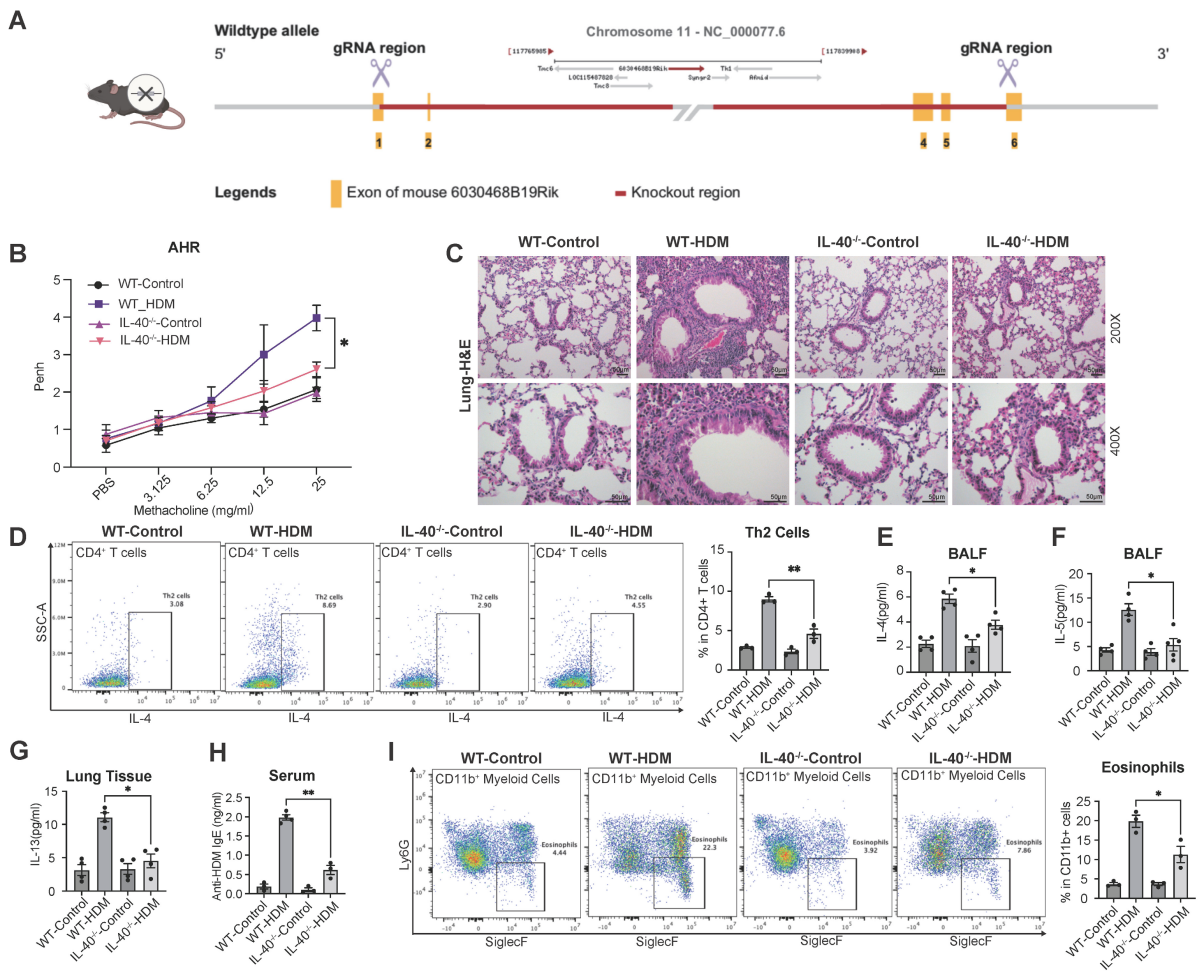
** IL-40 knockout alleviates HDM-induced asthma symptoms in mice. A** Generation of IL-40 knockout (IL-40^-/-^) mice by CRISPR-Cas9 technology. **B** AHR to methacholine challenge in wild-type (WT) and IL-40^-/-^ mice with or without HDM treatment (n=5). **C** Representative H&E-stained lung tissue sections. **D** Th2 cells were defined as IL-4^+^ cells within the CD45^+^CD3^+^CD4^+^ population in lung tissue, and representative flow cytometry plots and quantification are shown (n=3). **E, F** Concentrations of IL-4 and IL-5 in serum and BALF (n=4-6) measured by ELISA, respectively. **G** Concentration of IL-13 in lung tissue homogenates (n=4) measured by ELISA (n=4). **H** Concentration of anti-HDM IgE (n=3-4) in mouse serum measured by ELISA. **I** Eosinophils were defined as Ly-6G^-^SiglecF^+^ cells within the CD11b^+^ population in BALF, and representative flow cytometry plots and quantification are shown (n=3). Data are representative of at least three individual experiments. Values are expressed as the mean ± SEM. *P < 0.05; **P < 0.01 by Mann-Whitney test.

To elucidate the functional mechanisms of IL-40 in allergic asthma, we designed an IL-40^-/-^ knockout mice model with CRISPR-Cas9 technology (Fig. 3A). IL-40^-/-^ mice exhibited normal development and gene knockout was confirmed at the DNA and mRNA levels.

Upon HDM challenge, IL-40^-/-^ mice were markedly protected from developing asthma phenotypes. Compared with wild-type controls, IL-40^-/-^ mice exhibited significantly attenuated AHR (Fig. 3B) and reduced pulmonary inflammation (Fig. 3C). Flow cytometric analysis revealed fewer Th2 cells in lung tissue of IL-40^-/-^ mice after HDM exposure (Fig. 3D). Consistently, the levels of IL-4 and IL-5 in BALF (Fig. 3E, 3F) and IL-13 in lung homogenates (Fig. 3G) were all significantly decreased in IL-40^-/-^ mice relative to WT asthmatic mice. The serum level of anti-HDM IgE was also reduced (Fig. 3H). After HDM challenge, total cell numbers in BALF were significantly increased in WT mice, whereas the increase was attenuated in IL-40^-/-^ mice (Supplementary Fig. 1E). Moreover, flow cytometry showed that the proportion of eosinophils in BALF was markedly lower in IL-40^-/-^ mice (Fig. 3I). This phenotype was consistent with the asthma-mitigating effects observed with abIL-40. Supporting these findings, RNA-seq data demonstrated that IL-40 deficiency alters basal immune gene expression in the lungs without affecting normal physiological status (Supplementary Fig. 1F). Notably, Th17 cell expansion was also reduced in IL-40^-/-^ mice following HDM challenge (Supplementary Fig. 1G). IL-40 is therefore important for the development of immune response in allergic asthma.

### Deletion of IL-40 affected the abundance of B cell populations

**Figure 4:**
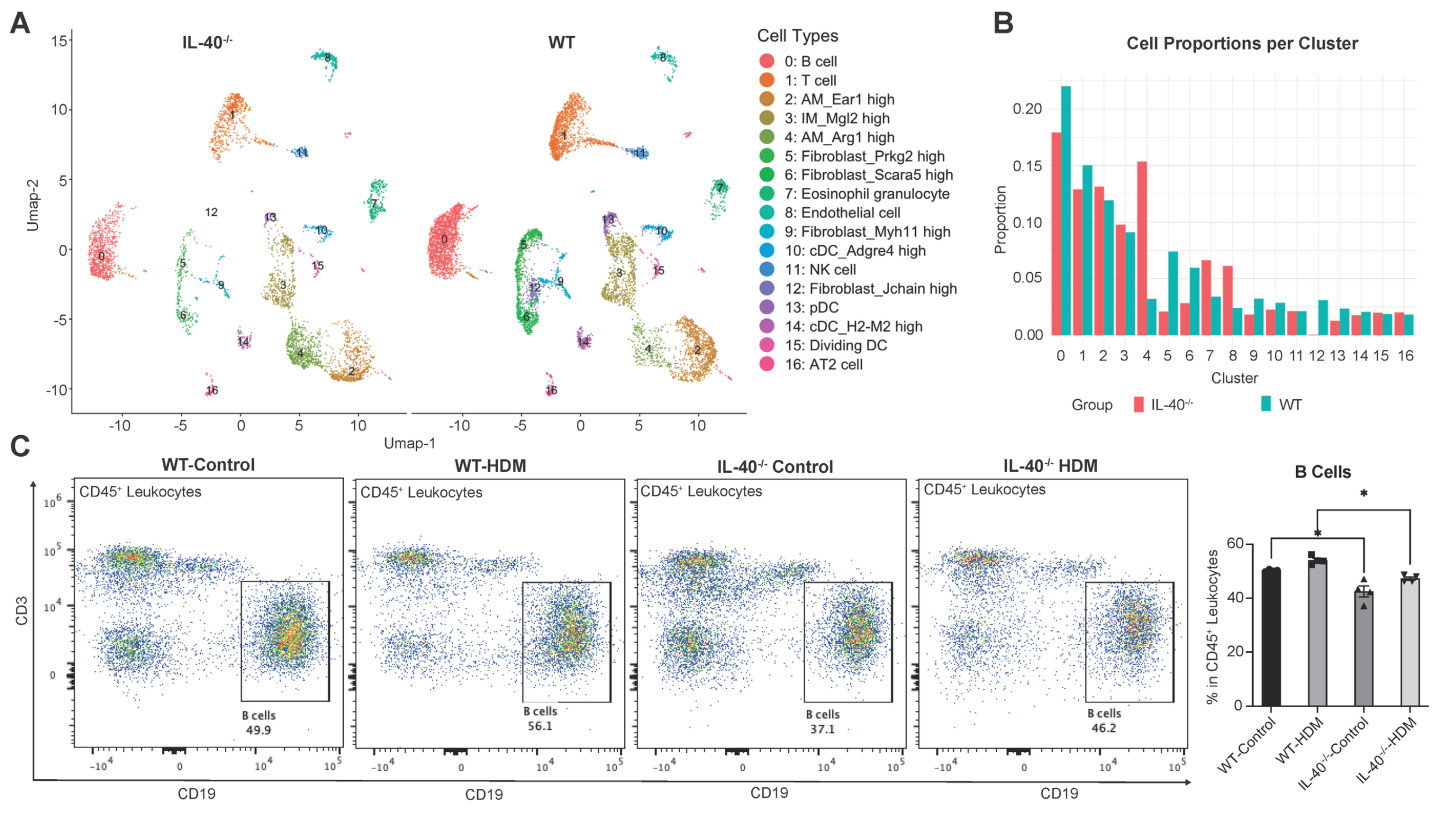
** IL-40 deletion alters the immune cell subsets in mouse lungs. A** UMAP visualization of annotated cell clusters in lung single-cell suspensions from IL-40^-/-^ and wild-type (WT) mice. **B** Relative proportions of each cell cluster among spleen cells for IL-40^-/-^ and WT mice. **C** B cells were defined as CD19^+^CD3^-^ cells within the CD45^+^ population in spleen, and representative flow cytometry plots and quantification are shown (n=4). Data are representative of at least three individual experiments. Values are expressed as the mean ± SEM. *P < 0.05 by Mann-Whitney test.

Multiple immune cell types contribute to the chronic inflammatory milieu in allergic asthma 27. To comprehensively characterize how IL-40 deficiency alters the pulmonary immune landscape, we performed scRNA-seq on lung tissues from both wild-type (WT) and IL-40^-/-^ mice (Fig. 4A, Supplementary Fig. 2A, Table S3).

Upon HDM stimulation, UMAP showed that the proportion of total B cells was significantly reduced in IL-40^-/-^ mice (0.17) compared to wild-type controls (0.22) (Fig. 4B). This reduction was also observed in spleen by flow cytometric analysis of CD45^+^CD19^+^ B cells (Fig. 4C), demonstrating that IL-40 is required for maintaining normal B cell abundance during allergic inflammation.

Gene ontology (GO) analysis indicated that B cells from IL-40^-/-^ mice exhibited downregulation of ribosome-associated pathways (Supplementary Fig. 2B), which could impair protein synthesis and hinder B cell differentiation and antibody production 28, 29, which may partially explain the reduced B cell abundance observed in the absence of IL-40.

### Significant expansion of Arg^+^ alveolar macrophages in the inflammatory environment following IL-40 knockout

**Figure 5:**
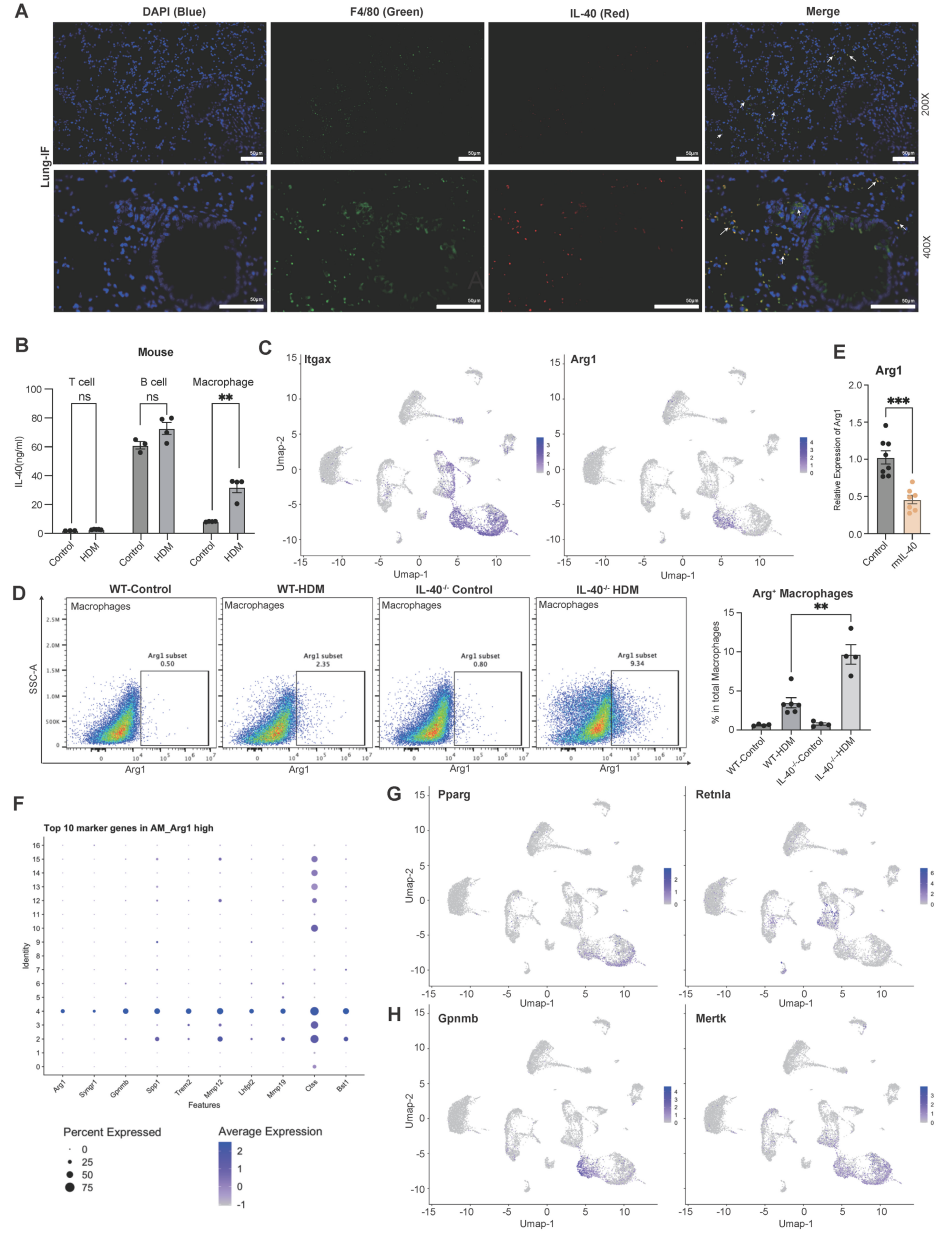
** IL-40 regulates the alterations in pulmonary macrophage subsets. A** Co-immunofluorescence staining of lung sections from WT asthmatic mice showing DAPI (blue), anti-F4/80 (green), and anti-IL-40 (red). Colocalization of IL-40 with F4/80⁺ macrophages is indicated by arrows. **B** Concentrations of IL-40 in cell lysates from mouse T cells (thymus), B cells (spleen), and macrophages (bone marrow) measured by ELISA (n=3-4). **C** ScRNA-seq analysis of *Itgax* and *Arg1* mRNA expression in different pulmonary cell types. **D** Macrophages were gated as F4/80^+^ cells within the CD11b^+^ population in lung tissue, and Arg1^+^ macrophages were identified; representative dot plots and quantification of Arg1^+^ macrophages as a percentage of total macrophages are shown (n=4-6). **E** Expression level of *Arg1* mRNA in mouse BMDM (n=8) measured by qRT-PCR. **F** Top 10 marker genes of the AM_Arg1 high macrophage cluster. **G** ScRNA-seq analysis of *Pparg* and *Retnla* mRNA expression in different cell types. **H** ScRNA-seq analysis of *Gpnmb* and *Mertk* mRNA expression in different cell types. Data are representative of at least three individual experiments. Values are expressed as the mean ± SEM. **P < 0.01, ***P < 0.001, and ns for not significant by Mann-Whitney test.

Consistent with findings from synovial samples from RA patients, our immunofluorescence results of mouse lung tissue revealed significant colocalization of IL-40 with macrophages (Fig. 5A). ELISA further confirmed that, compared with T and B cells, macrophages display the highest induction of IL-40 production following HDM stimulation (Fig. 5B).

ScRNA sequencing analysis further identified three distinct macrophage subpopulations in the lung: Alveolar Macrophage_*Ear1* high (AM_*Ear1* high), Interstitial Macrophage_*Mnl2* high (IM_*Mnl2* high), and Alveolar Macrophage_*Arg1* high (AM_*Arg1* high) (Fig. 4A, Fig. 5C). Notably, the AM_*Arg1* high subset was dramatically expanded in IL-40^-/-^ mice after HDM exposure, accounting for 15% of total lung cells versus only 3% in WT controls (Fig. 4B). This increase in *Arg1*^+^ macrophages upon IL-40 deletion was further validated by flow cytometry (Fig. 5D). *In vitro*, rmIL-40 stimulation of BMDMs suppressed *Arg1* expression, demonstrating a direct inhibitory effect (Fig. 5E).

Arg1 plays a key role in immune regulation by suppressing T cell proliferation and nitric oxide production, and exerting anti-inflammatory effects 30. Interestingly, despite the classical association of M2 polarization with enhanced Th2 inflammation 31, loss of IL-40 leads to both a reduction in overall airway inflammation and a marked increase in *Arg1*^+^ macrophages. Further analysis showed these cells lacked expression of other typical M2 markers such as *Cd206*, *Pparg*, and *Retnla*, but instead expressed genes such as *Gpnmb* and *Mertk* (Fig. 5F-H). GPNMB has showed efficacy in suppressing T cell activity 32, 33, and macrophage-MerTK maintains lung immune homeostasis by facilitating the clearance of apoptotic cells in the airways 34. This suggests that these *Arg1*^+^ macrophages may have immunoregulatory or tissue repair functions rather than promoting Th2-driven inflammation. Moreover, this notion is supported by previous reports that *Arg1*^+^ macrophages do not always exacerbate allergic asthma, especially in female mice, and that the deletion of IL-4Rα or *Arg1*, does not necessarily determine asthma severity or AHR 35-37. Taken together, these results indicate that IL-40 critically shapes the composition and function of pulmonary macrophage subsets during allergic inflammation.

### IL-40 modulates macrophage polarization by promoting pro-inflammatory phenotypes and selectively inhibiting *Arg1*^+^ M2-like subsets *in vitro*

**Figure 6:**
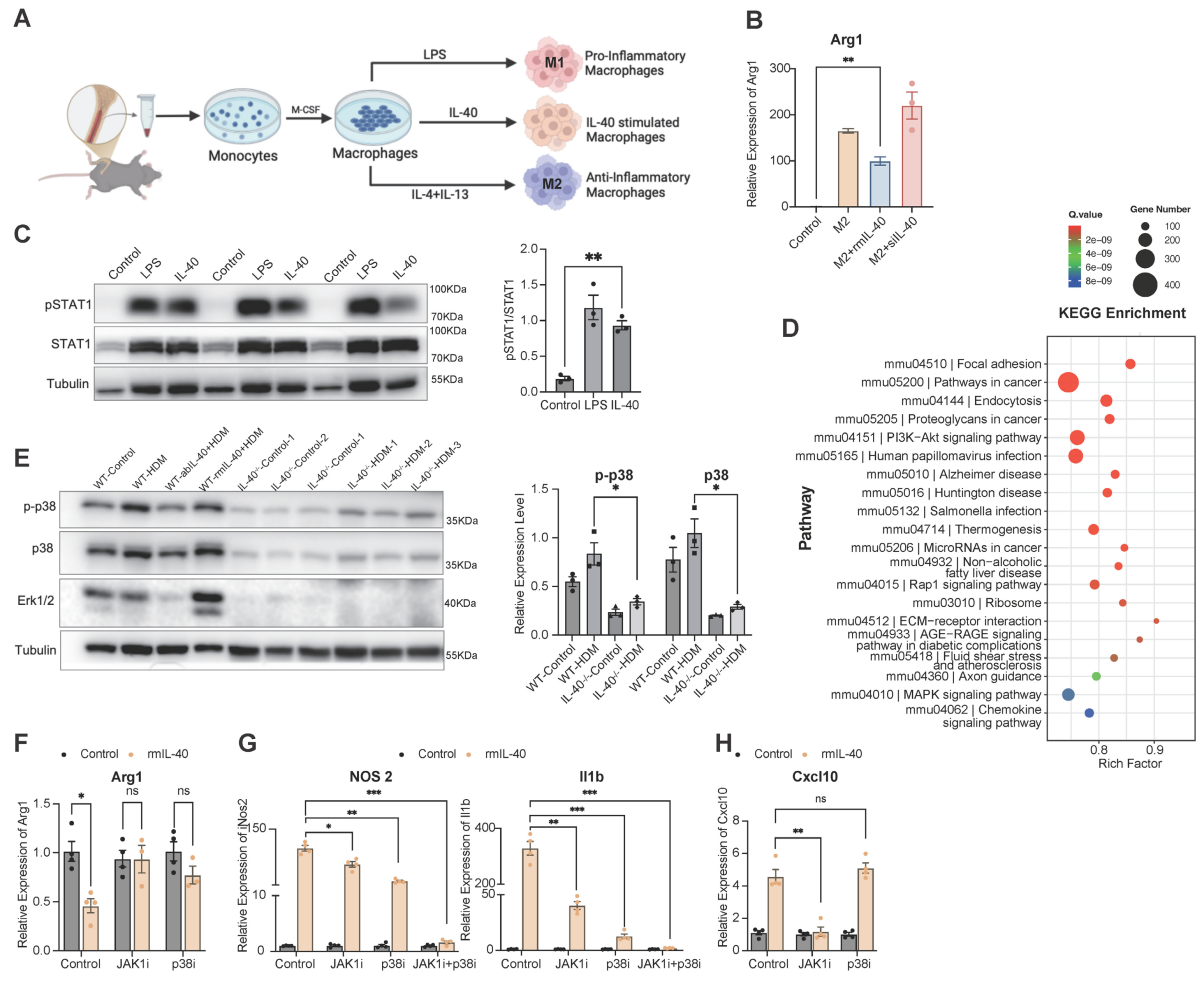
** IL-40 activates JAK-STAT1 and p38 pathways to regulate macrophage polarization. A** Schematic diagram of the experimental design for the isolation, culture, and stimulation of BMDMs. M1 macrophages were generated by stimulation with LPS, M2 macrophages were generated by stimulation with IL-4 and IL-13, and IL-40-stimulated BMDMs are shown in parallel for comparison. **B** Expression level of *Arg1* mRNA in treated BMDMs (n = 4) measured by qRT-PCR. **C** Western blot analysis of JAK-STAT1 pathway components (pSTAT1, STAT1) in treated wild-type BMDMs, quantification of pSTAT1/STAT1 ratio is shown (n = 3). **D** Gene Ontology (GO) and KEGG pathway analysis of differentially expressed genes (DEGs) from bulk RNA-seq of wild-type and IL-40^-/-^ mouse lung tissues. **E** Western blot analysis of p38-MAPK pathway activation (p-p38, p38) in BMDMs from IL-40^-/-^ mice and wild-type controls upon stimulation; quantification of relative p-p38 expression levels is shown (n = 3). **F-H** Expression level of *Arg1* (F), *NOS2* and *Il1b* (G), and *Cxcl10* (H) in treated BMDMs (n=4-5) measured by qRT-PCR. Data are representative of at least three individual experiments. Values are expressed as the mean ± SEM. *P < 0.05, **P < 0.01, ***P < 0.001, and ns for not significant by Mann-Whitney test.

To further elucidate the molecular mechanisms by which IL-40 regulates macrophage polarization, we utilized an *in vitro* system with murine BMDMs (Fig. 6A). In wild-type mice, macrophages were induced into a pro-inflammatory M1-like state with high *NOS2* expression upon either lipopolysaccharide (LPS) or rmIL-40 treatment, however, in IL-40^-/-^ mice, while LPS could still induce M1 polarization, the exogenous supplementation of rmIL-40 failed to promote this similar change (Supplementary Fig. 3A), thereby suggesting possible adaptive rewiring of intracellular signaling after developmental loss of IL-40.

In addition, we found that supplementation with rmIL-40 selectively suppressed the expression of certain M2-associated genes, such as *Arg1* (Fig. 5E), while having little effect on other classical M2 markers such as *Cd206* (Supplementary Fig. 3B). These findings support that IL-40 selectively inhibits the polarization of a *Arg1* high macrophage subset, rather than broadly suppressing all M2-associated genes.

When BMDMs had already reached a fully polarized state, subsequent IL-40 addition did not further enhance M1 gene expression (Supplementary Fig. 3C) and only partially suppressed *Arg1* in M2-polarized cells (Fig. 6B), indicating that IL-40's regulatory capacity is mostly manifest during the early polarization process.

### IL-40 stimulates the activation of the JAK-STAT1 and p38 pathways to influence macrophage polarization

To further dissect the cellular targets and downstream signaling of IL-40 in allergic inflammation, we isolated and stimulated mouse primary T cells, macrophages, with rmIL-40 across multiple time points. Among these, macrophages exhibited the most robust response to IL-40 stimulation, as demonstrated by early activation of significant phenotypic changes even at low IL-40 concentrations (Supplementary Fig. 4A), whereas T cells were almost unresponsive after 12 hours (Supplementary Fig. 4B). Similarly, primary human macrophages also responded to IL-40 stimulation (Supplementary Fig. 4C), and neither T cells nor bronchial epithelial cells showed direct response at 12h point (Supplementary Fig. 4D, E). However, co-culture assays revealed that IL-40-primed macrophages could augment Th2 cytokine like IL-13 and IL-4 production in T cells, indicating that IL-40 indirectly influences T cell function through macrophage-mediated mechanisms (Supplementary Fig. 4F).

At the mechanistic level, bulk RNA-seq analysis of mouse lung tissue showed that HDM stimulation upregulated JAK-STAT1 pathway genes such as *Stat1* and *Stat2* in wild-type mice, but this response was attenuated in IL-40^-/-^ mice (Supplementary Fig. 4G). Consistently, stimulation of BMDMs with rmIL-40 led to pronounced activation of STAT1 (Fig. 6C), supporting a direct role for IL-40 in activating the JAK/STAT1 cascade 38. GO analysis further indicated that downregulated genes of lung tissues in IL-40^-/-^ mice were significantly enriched in the MAPK pathway 39 (Fig. 6D). Furthermore, levels of p38 and Erk1/2 were markedly decreased in the lungs of IL-40^-/-^ mice and could not be activated by HDM induction (Fig. 6E). Similarly, exogenous IL-40 could not restore p38-MAPK activation in IL-40^-/-^ BMDMs (Supplementary Fig. 4H).

Functional interrogation using specific inhibitors JAK1i (JAKi) and p38i (SB203580) in BMDMs (Supplementary Fig. 4I, J) revealed that either inhibition could partially reduce the ability of IL-40 to promote macrophage polarization, and combined inhibition markedly reduced this effect, as shown by decreased M1 marker *NOS2* and *Il1b* expression and increased *Arg1* (Fig. 6F, G). For *Cxcl10*, a gene primarily regulated by the JAK/STAT1 pathway, JAK inhibition significantly diminished the IL-40 effect, whereas p38 inhibition had minimal influence (Fig. 6H). Additionally, siRNA-mediated knockdown of IL-40 in RAW264.7 macrophages led to a profound loss of p38 signaling (Supplementary Fig. 4K). These results demonstrate that IL-40 acts upstream to co-activate both the p38/MAPK and JAK/STAT1 pathways, modulating macrophage polarization.

### IL-40 regulates early differentiation and maturation of B cells in bone marrow and spleen

**Figure 7:**
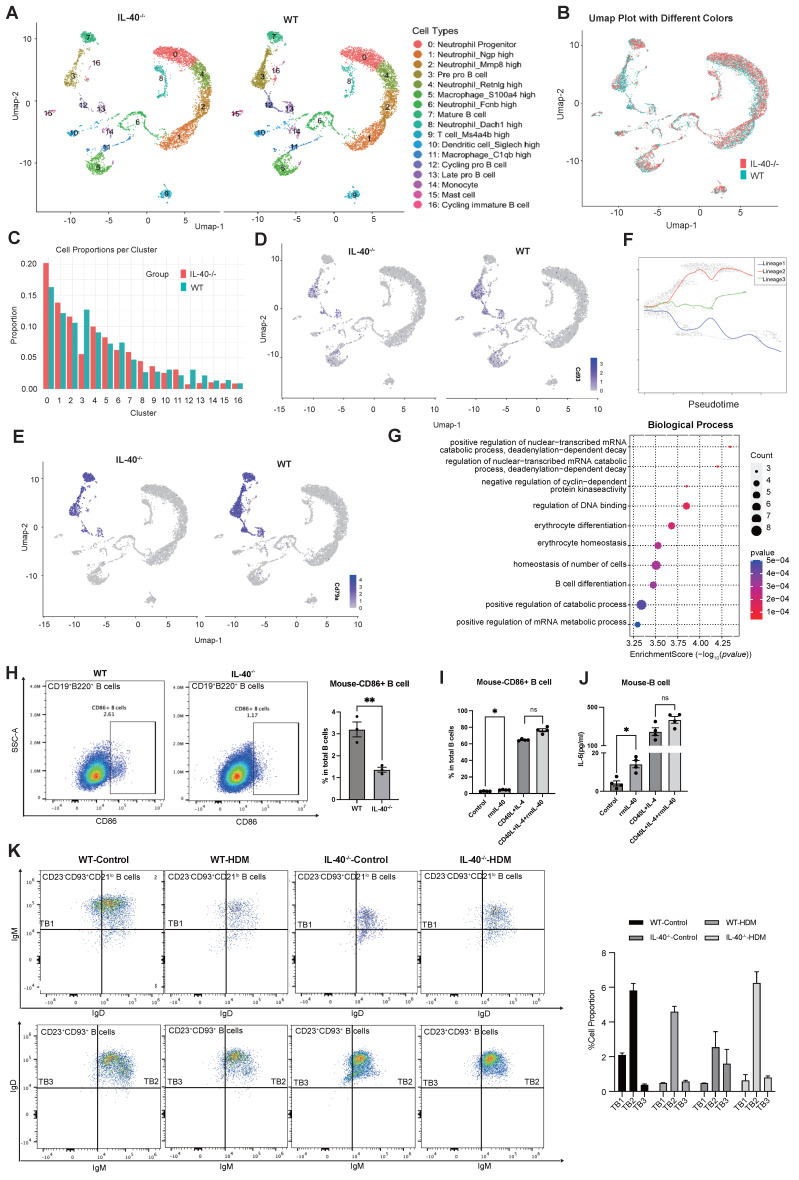
** IL-40 deficiency inhibits the development and differentiation of B cells in bone marrow. A** UMAP visualization of annotated cell type clusters in bone marrow from IL-40^-/-^ and wild-type (WT) mice by single-cell RNA sequencing. **B** UMAP plot showing distribution of clusters from IL-40^-/-^ and WT mice. **C** Quantification of the percentage of each cell cluster in bone marrow from IL-40^-/-^ and WT mice. **D, E** ScRNA-seq analysis of *Cd93* and *Cd79a* mRNA expression in mouse bone marrow. **F** Pseudotime trajectory analysis of B cell differentiation in bone marrow using Slingshot. **G** Gene Ontology (GO) enrichment analysis of differentially expressed genes (DEGs) in all B cell populations between IL-40^-/-^ and WT mice. **H, I** CD86+ B cells were gated within the CD19^+^B220^+^ population in B cells isolated from mouse spleen, representative dot plots and quantification of CD86^+^ B cells as a percentage of total B cells are shown (n=4-6). **J** Concentrations of IL-6 in cell lysates from mouse B cells measured by ELISA (n=3-4). **K** Flow cytometry analysis of splenic B cell transitional subsets, B cells were identified as CD19^+^ and further transitional B cells were classified as TB1 (CD23^-^CD93^+^CD21^+/lo^IgM^hi^IgD^-/lo^), TB2 (CD23^+^CD93^+^CD21^lo^IgM^hi^IgD^hi^), and TB3 (CD23^+^CD93^+^CD21^lo^IgM^lo^IgD^hi^), and the proportion of each subset is shown in the accompanying bar graph. Data are representative of at least three individual experiments. Values are expressed as the mean ± SEM.

Given the high expression of IL-40 in fetal liver and its tight association with B cells, IL-40 may also play a role in the early B cell development, we performed scRNA sequencing of bone marrow from wild-type and IL-40^-/-^ mice (Fig. 7A, Table S4). UMAP and clustering analysis revealed a marked reduction in all four B cell subpopulations upon IL-40 deficiency, with the total B cell pool decreased by more than 50% (Fig. 7B, C). Cluster 3 (pre-pro B cell) showed the most pronounced decrease. *Cd93*, a key regulator of BCR synthesis, pre-pro-B cell survival and plasma cell differentiation and an activator of the p38 MAPK pathway 40-42, was markedly downregulated in IL-40^-/-^ mice, especially in cluster 3, 12 and 13 (Fig. 7D). Similarly, *Cd79a*, essential for early B cell development and pre-BCR signaling 43, was also significantly reduced in the absence of IL-40 (Fig. 7E). Pseudotime analysis revealed a trajectory from cluster 3 through cluster 12 to cluster 13 (Fig. 7F), and the positioning of cluster 3 at the root of this trajectory, together with its early B cell marker profile, further confirms its identity as an early B cell subset. Enrichment analysis of differentially expressed genes further confirmed a reduction in gene sets related to B cell differentiation in IL-40^-/-^ bone marrow (Fig. 7G).

To directly assess the role of IL-40 in B cell function, we isolated B cells from mouse spleen and human PBMCs for *in vitro* stimulation. Notably, mouse B cells lacking IL-40 displayed significantly reduced CD86 expression compared to wild-type controls (Fig. 7H) at 24h point, suggesting that sustained IL-40 deficiency may lead to adaptive changes in B cell activation potential. When B cells were treated with IL-40, we observed a modest increase in CD86 expression in both species (Fig. 7I, Supplementary Fig. 5A, B) after 24 hours. The addition of IL-40 to CD40L and IL-4 co-stimulation resulted in only a mild enhancement of CD86 and IL-6 production, although not always statistically significant (Fig. 7J, Supplementary Fig. 5C). Together, these findings indicate that IL-40 can directly contribute to B cell activation and cytokine induction.

Spleen is a crucial secondary lymphoid organ that supports the maturation, activation, and differentiation of B cells 44. To address the effects of IL-40 on peripheral B cell maturation, we further analyzed splenic B cell subsets by flow cytometry. Consistent with bone marrow findings, IL-40^-/-^ mice exhibited a decrease in mature follicular B cells, with significant reductions in transitional B cell 3 (TB3; CD21^lo^IgM^lo^IgD^hi^) and a relative accumulation of TB2 (CD21^lo^IgM^hi^IgD^hi^) 44. Upon HDM challenge, wild-type mice showed a normal shift toward TB3 as part of the adaptive immune response, whereas IL-40^-/-^ mice exhibited impaired maturation from TB2 to TB3, suggesting a developmental blockade (Fig. 7K). Collectively, these results demonstrate that IL-40 critically regulates early differentiation and maturation of B cells in both bone marrow and spleen. Loss of IL-40 leads to defective B cell development, diminished B cell activation and antigen presentation.

### IL-40 regulates the complement system to promote B cell differentiation

Given the established relationship between IL-40 and both B cell differentiation and macrophage polarization, we further investigated transcriptomic changes in the lung following HDM induction. In IL-40^-/-^ mice, many of the downregulated DEGs in lung tissue were significantly enriched in complement-related pathways, including classical antibody-mediated complement activation (Supplementary Fig. 6A). The complement system plays a key role in enhancing BCR signaling by binding complement fragments such as C3d to complement receptor 2 (CR2/CD21) on B cells, thus forming a co-receptor complex that lowers the activation threshold of B cells and promotes their activation, proliferation, and antibody production 45, 46. Consistently, qRT-PCR confirmed that complement-related genes were downregulated in lung tissue after IL-40 deletion, while *in vitro* stimulation with rmIL-40 significantly activated complement system gene expression (Supplementary Fig. 6B). These findings suggest that IL-40 may regulate B cell differentiation and development by modulating the complement pathway and facilitating BCR synthesis.

## Discussion

**Figure 8:**
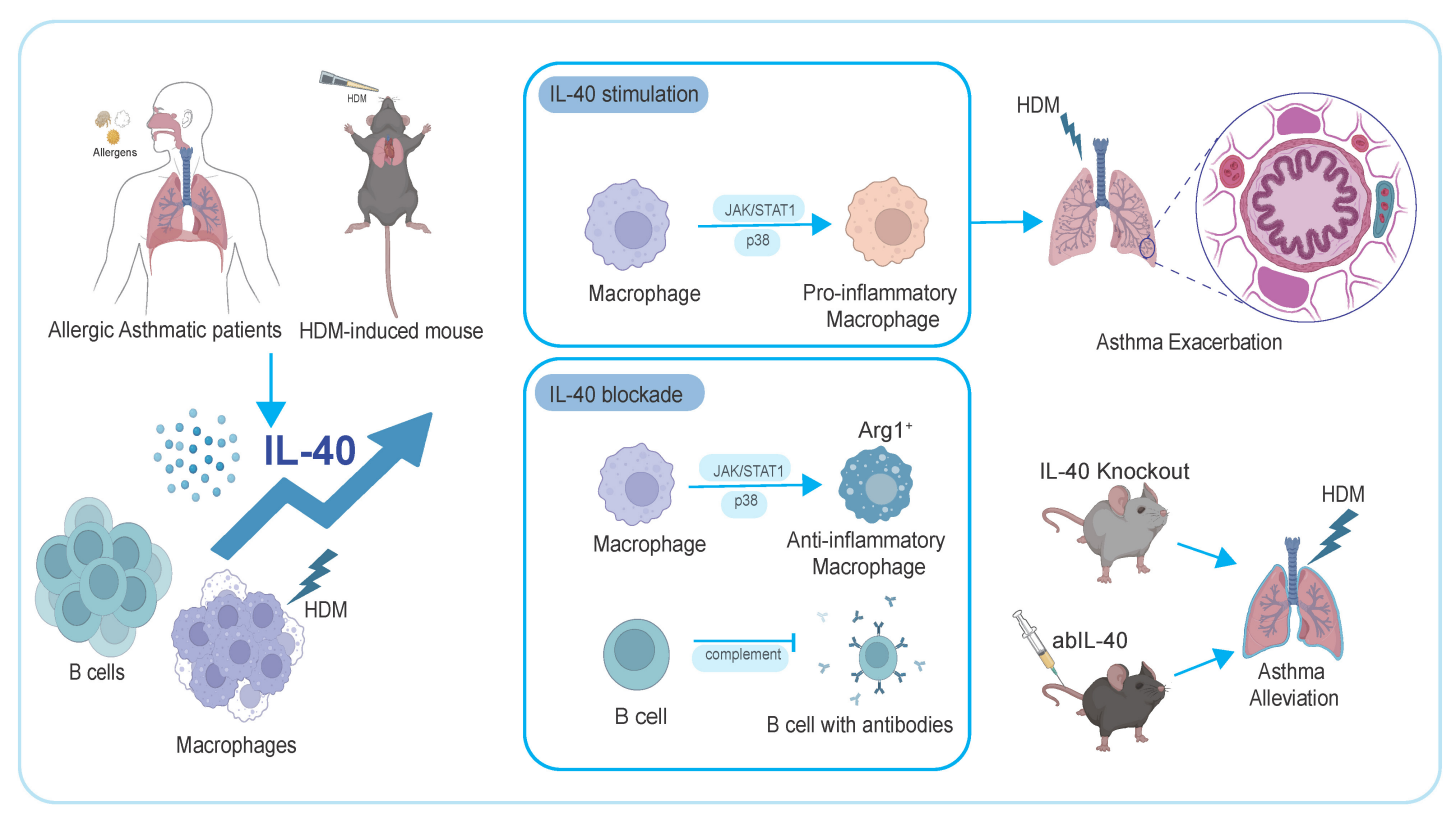
** Summary diagram of IL-40 function in allergic asthma.** IL-40 is upregulated in allergic asthmatic patients and HDM-induced asthmatic mice. It promotes airway inflammation by driving pro-inflammatory macrophage polarization via JAK/STAT1 and p38-MAPK signaling, and facilitating B cell development. Neutralizing IL-40 or knocking it out reduces airway inflammation and alleviates asthma symptoms, highlighting its key immunological role.

Cytokines secreted by B cells are limited in amount but play pivotal roles in immune regulation. Regulatory B cells secrete IL-10, which suppresses Th2 activation and alleviates airway inflammation in allergic asthma 47. IL-40, a recently identified cytokine secreted by B cells, has been implicated in several immune-mediated conditions 20, including RA 22, sepsis 24 and primary Sjögren's syndrome 48. Recent evidence indicates that IL-40 regulates inflammatory responses by interacting with neutrophils and other immune cell subsets 24, 49. However, the specific pathways and cellular targets of IL-40 remain largely undefined. Our study provides new insights into these mechanisms, thereby advancing the understanding of IL-40 in immune regulation and disease pathology (Fig. 8).

Our findings identify IL-40 as a key immunoregulatory cytokine in allergic asthma, produced predominantly by B cells together with macrophages, especially following HDM exposure. IL-40 promotes Th2-type immune responses and eosinophilic infiltration, thereby exacerbating hallmark features of asthma. Both genetic deletion and neutralization of IL-40 alleviate these pathological features, supporting its role as an active participant in allergic inflammation rather than a mere biomarker.

Allergic asthma is increasingly recognized as a heterogeneous disease, with a subset of patients exhibiting mixed granulocytic inflammation often linked to increased severity and steroid resistance 50-52. Beyond its effects on Th2 responses, our data show that IL-40 drives M1-like macrophage polarization. Notably, M1-polarized macrophages are known to secrete pro-inflammatory mediators that can enhance neutrophil recruitment and activation, thereby contributing to neutrophil extracellular traps-mediated cell death (NETosis) 53-55. This connection is particularly relevant as recent work in sepsis and RA has shown that IL-40 deficiency suppresses NETosis, which leads to attenuated neutrophil-driven inflammation and improved disease outcomes 24. Therefore, IL-40's ability to modulate both macrophage polarization and neutrophil function positions it as a promising upstream target for mixed-type asthma, where granulocytic inflammation drives disease progression and complicates treatment response.

Importantly, our results reveal that the expansion of *Arg1^+^* macrophages in IL-40-deficient mice is paradoxically associated with reduced airway inflammation. This observation suggests that IL-40 can regulate the balance between distinct macrophage populations 56. Because these Arg1^+^ macrophages lack other classical M2 markers, they may instead possess immunoregulatory or tissue repair functions 37. The ability of IL-40 to skew macrophage polarization underscores its central role in shaping the inflammatory landscape of the asthmatic lung.

IL-40 is critical for the maintenance and maturation of B cells. Loss of IL-40 disrupts B cell homeostasis, impairs humoral immune responses, and alters the pulmonary immune microenvironment. Downregulation of key genes such as *Cd93* and *Cd79a* further implicates IL-40 in the regulation of early B cell development and maturation, our data also indicate that IL-40 modulates the complement pathway 57-59. These findings suggest that IL-40 is a vital link between innate and adaptive immunity in the context of allergic airway inflammation.

Despite these promising findings, several questions remain to be addressed. For example, the precise mechanisms by which IL-40 regulates the balance among macrophage subsets warrant further investigation. While our data support a key role for IL-40 in B cell development and function, the downstream pathways linking IL-40 to B cell receptor signaling and complement activation merit deeper exploration. Furthermore, future studies should assess the safety and efficacy of IL-40-targeted therapies both in preclinical models and in clinical trials. Given the complex interplay between innate and adaptive immunity in asthma, combination therapies that incorporate IL-40 inhibition with established biologics may provide superior control for patients with severe or treatment-resistant disease 60.

To conclude, these insights position IL-40 as a promising target for clinical intervention and biomarker development in asthma. The identification of IL-40 as a central mediator of both humoral and innate immunity offers opportunities for improved patient stratification and the development of targeted biologics 61-63. IL-40 inhibitors, either as monotherapy or in combination with existing treatments, hold the potential to address the unmet needs of patients with refractory or complex asthma endotypes. Ultimately, advancing our understanding of IL-40-mediated pathways may pave the way for novel therapeutic strategies tailored to address the immunological heterogeneity observed in asthma, thereby improving patient outcomes in clinical practice.

## Supplementary Material

Supplementary figures and tables 1-2.

Supplementary table 3: bone marrow scRNAseq markers.

Supplementary table 4: lung scRNAseq markers.

## References

[1] Holgate ST, Wenzel S, Postma DS, Weiss ST, Renz H, Sly PD (2015). Asthma. Nat Rev Dis Primers.

[2] Collaborators GAaAD (2025). Global, regional, and national burden of asthma and atopic dermatitis, 1990-2021, and projections to 2050: a systematic analysis of the Global Burden of Disease Study 2021. Lancet Respir Med.

[3] Schatz M, Rosenwasser L (2014). The allergic asthma phenotype. J Allergy Clin Immunol Pract.

[4] Sears MR, Herbison GP, Holdaway MD, Hewitt CJ, Flannery EM, Silva PA (1989). The relative risks of sensitivity to grass pollen, house dust mite and cat dander in the development of childhood asthma. Clin Exp Allergy.

[5] Hammad H, Lambrecht BN (2021). The basic immunology of asthma. Cell.

[6] Froidure A, Mouthuy J, Durham SR, Chanez P, Sibille Y, Pilette C (2016). Asthma phenotypes and IgE responses. Eur Respir J.

[7] Agache I, Beltran J, Akdis C, Akdis M, Canelo-Aybar C, Canonica GW (2020). Efficacy and safety of treatment with biologicals (benralizumab, dupilumab, mepolizumab, omalizumab and reslizumab) for severe eosinophilic asthma. A systematic review for the EAACI Guidelines - recommendations on the use of biologicals in severe asthma. Allergy.

[8] Akenroye AT, Segal JB, Zhou G, Foer D, Li L, Alexander GC (2023). Comparative effectiveness of omalizumab, mepolizumab, and dupilumab in asthma: A target trial emulation. J Allergy Clin Immunol.

[9] Castro M, Corren J, Pavord ID, Maspero J, Wenzel S, Rabe KF (2018). Dupilumab Efficacy and Safety in Moderate-to-Severe Uncontrolled Asthma. N Engl J Med.

[10] Ortega HG, Liu MC, Pavord ID, Brusselle GG, FitzGerald JM, Chetta A (2014). Mepolizumab treatment in patients with severe eosinophilic asthma. N Engl J Med.

[11] Arasi S, Cafarotti A, Galletta F, Panetta V, Riccardi C, Calandrelli V (2025). Omalizumab reduces anaphylactic reactions and allows food introduction in food-allergic in children with severe asthma: An observational study. Allergy.

[12] Ogulur I, Mitamura Y, Yazici D, Pat Y, Ardicli S, Li M (2025). Type 2 immunity in allergic diseases. Cell Mol Immunol.

[13] Nelson AJ, Tatematsu BK, Beach JR, Sojka DK, Wu YL (2025). Lung-resident memory B cells maintain allergic IgE responses in the respiratory tract. Immunity.

[14] Wang SR, Hu RD, Ma M, You X, Cui H, He Y (2022). FoxO1 suppresses IL-10 producing B cell differentiation via negatively regulating Blimp-1 expression and contributes to allergic asthma progression. Mucosal Immunol.

[15] Britt RD Jr, Ruwanpathirana A, Ford ML, Lewis BW (2023). Macrophages Orchestrate Airway Inflammation, Remodeling, and Resolution in Asthma. Int J Mol Sci.

[16] Zhong Y, Huang T, Huang J, Quan J, Su G, Xiong Z (2023). The HDAC10 instructs macrophage M2 program via deacetylation of STAT3 and promotes allergic airway inflammation. Theranostics.

[17] Tee JH, Vijayakumar U, Shanmugasundaram M, Lam TYW, Liao W, Yang Y (2023). Isthmin-1 attenuates allergic Asthma by stimulating adiponectin expression and alveolar macrophage efferocytosis in mice. Respir Res.

[18] Wang Y, Wernersbach I, Strehle J, Li S, Appel D, Klein M (2022). Early posttraumatic CSF1R inhibition via PLX3397 leads to time- and sex-dependent effects on inflammation and neuronal maintenance after traumatic brain injury in mice. Brain Behav Immun.

[19] Gao X, Leung TF, Wong GW, Ko WH, Cai M, He EJ (2022). Meteorin-β/Meteorin like/IL-41 attenuates airway inflammation in house dust mite-induced allergic asthma. Cell Mol Immunol.

[20] Dabbagh-Gorjani F (2024). A Comprehensive Review on the Role of Interleukin-40 as a Biomarker for Diagnosing Inflammatory Diseases. Autoimmune Dis.

[21] Catalan-Dibene J, Vazquez MI, Luu VP, Nuccio SP, Karimzadeh A, Kastenschmidt JM (2017). Identification of IL-40, a Novel B Cell-Associated Cytokine. J Immunol.

[22] Wang W, Zhao J, Wu S, Fu J, Zhang Y, Peng W (2024). Serum IL-40 increases in patients with rheumatoid arthritis and correlates with some clinical characteristics and comorbidities. Sci Rep.

[23] Cerezo LA, Navrátilová A, Kuklová M, Prokopcová A, Baloun J, Kropáčková T (2024). IL-40 is up-regulated in the synovial fluid and cartilage of osteoarthritis patients and contributes to the alteration of chondrocytes phenotype in vitro. Arthritis Res Ther.

[24] Cai S, Li X, Zhang C, Jiang Y, Liu Y, He Z (2025). Inhibition of Interleukin-40 prevents multi-organ damage during sepsis by blocking NETosis. Crit Care.

[25] Navrátilová A, Andrés Cerezo L, Hulejová H, Bečvář V, Tomčík M, Komarc M (2021). IL-40: A New B Cell-Associated Cytokine Up-Regulated in Rheumatoid Arthritis Decreases Following the Rituximab Therapy and Correlates With Disease Activity, Autoantibodies, and NETosis. Front Immunol.

[26] Saraiva M, O'Garra A (2010). The regulation of IL-10 production by immune cells. Nat Rev Immunol.

[27] Komlósi ZI, van de Veen W, Kovács N, Szűcs G, Sokolowska M, O'Mahony L (2022). Cellular and molecular mechanisms of allergic asthma. Mol Aspects Med.

[28] Xu J, Zhong A, Zhang S, Chen M, Zhang L, Hang X (2023). KMT2D Deficiency Promotes Myeloid Leukemias which Is Vulnerable to Ribosome Biogenesis Inhibition. Adv Sci (Weinh).

[29] Romijn EP, Christis C, Wieffer M, Gouw JW, Fullaondo A, van der Sluijs P (2005). Expression clustering reveals detailed co-expression patterns of functionally related proteins during B cell differentiation: a proteomic study using a combination of one-dimensional gel electrophoresis, LC-MS/MS, and stable isotope labeling by amino acids in cell culture (SILAC). Mol Cell Proteomics.

[30] Haydar D, Gonzalez R, Garvy BA, Garneau-Tsodikova S, Thamban Chandrika N, Bocklage TJ (2021). Myeloid arginase-1 controls excessive inflammation and modulates T cell responses in Pseudomonas aeruginosa pneumonia. Immunobiology.

[31] Abdelaziz MH, Abdelwahab SF, Wan J, Cai W, Huixuan W, Jianjun C (2020). Alternatively activated macrophages; a double-edged sword in allergic asthma. J Transl Med.

[32] Saade M, Araujo de Souza G, Scavone C, Kinoshita PF (2021). The Role of GPNMB in Inflammation. Front Immunol.

[33] Masand N, Perry TA, Pugh M, Fennell E, Hennessy A, Wei W (2025). Hodgkin/Reed-Sternberg cells induce GPNMB expression and release from macrophages to suppress T-cell responses to the Epstein-Barr virus-encoded LMP2A protein. Haematologica.

[34] Guimarães-Pinto K, Leandro M, Corrêa A, Maia EP, Rodrigues L, da Costa ALA (2024). Differential regulation of lung homeostasis and silicosis by the TAM receptors MerTk and Axl. Front Immunol.

[35] Nieuwenhuizen NE, Kirstein F, Jayakumar J, Emedi B, Hurdayal R, Horsnell WG (2012). Allergic airway disease is unaffected by the absence of IL-4Rα-dependent alternatively activated macrophages. J Allergy Clin Immunol.

[36] Cloots RHE, Sankaranarayanan S, Poynter ME, Terwindt E, van Dijk P, Lamers WH (2017). Arginase 1 deletion in myeloid cells affects the inflammatory response in allergic asthma, but not lung mechanics, in female mice. BMC Pulm Med.

[37] Cloots RH, Sankaranarayanan S, de Theije CC, Poynter ME, Terwindt E, van Dijk P (2013). Ablation of Arg1 in hematopoietic cells improves respiratory function of lung parenchyma, but not that of larger airways or inflammation in asthmatic mice. Am J Physiol Lung Cell Mol Physiol.

[38] Ivashkiv LB (2018). IFNγ: signalling, epigenetics and roles in immunity, metabolism, disease and cancer immunotherapy. Nat Rev Immunol.

[39] Zhang X, Fan L, Wu J, Xu H, Leung WY, Fu K (2019). Macrophage p38α promotes nutritional steatohepatitis through M1 polarization. J Hepatol.

[40] Jiang K, Xu Y, Wang Y, Yin N, Huang F, Chen M (2025). Deciphering the role of IL-17D, its newly identified receptor CD93, and IL-17D-CD93 axis in health and disease. J Immunol.

[41] Chevrier S, Genton C, Kallies A, Karnowski A, Otten LA, Malissen B (2009). CD93 is required for maintenance of antibody secretion and persistence of plasma cells in the bone marrow niche. Proc Natl Acad Sci U S A.

[42] Ni X, Xu Y, Wang W, Kong B, Ouyang J, Chen J (2022). IL-17D-induced inhibition of DDX5 expression in keratinocytes amplifies IL-36R-mediated skin inflammation. Nat Immunol.

[43] Huse K, Bai B, Hilden VI, Bollum LK, Våtsveen TK, Munthe LA (2022). Mechanism of CD79A and CD79B Support for IgM+ B Cell Fitness through B Cell Receptor Surface Expression. J Immunol.

[44] Chung JB, Silverman M, Monroe JG (2003). Transitional B cells: step by step towards immune competence. Trends Immunol.

[45] Carroll MC (2004). The complement system in B cell regulation. Mol Immunol.

[46] Lyubchenko T, dal Porto J, Cambier JC, Holers VM (2005). Coligation of the B cell receptor with complement receptor type 2 (CR2/CD21) using its natural ligand C3dg: activation without engagement of an inhibitory signaling pathway. J Immunol.

[47] Qian G, Jiang W, Sun D, Sun Z, Chen A, Fang H (2023). B-cell-derived IL-10 promotes allergic sensitization in asthma regulated by Bcl-3. Cell Mol Immunol.

[48] Guggino G, Rizzo C, Mohammadnezhad L, Lo Pizzo M, Lentini VL, Di Liberto D (2023). Possible role for IL-40 and IL-40-producing cells in the lymphocytic infiltrated salivary glands of patients with primary Sjögren's syndrome. RMD Open.

[49] Navrátilová A, Bečvář V, Hulejová H, Tomčík M, Štolová L, Mann H (2023). New pro-inflammatory cytokine IL-40 is produced by activated neutrophils and plays a role in the early stages of seropositive rheumatoid arthritis. RMD Open.

[50] Simpson JL, Scott R, Boyle MJ, Gibson PG (2006). Inflammatory subtypes in asthma: assessment and identification using induced sputum. Respirology.

[51] Plavsic A, Bonaci-Nikolic B, Milenkovic B, Miskovic R, Kusic N, Dimitrijevic M (2024). Asthma Inflammatory Phenotypes: How Can We Distinguish Them?. J Clin Med.

[52] Humbert M, Menz G, Ying S, Corrigan CJ, Robinson DS, Durham SR (1999). The immunopathology of extrinsic (atopic) and intrinsic (non-atopic) asthma: more similarities than differences. Immunol Today.

[53] Zhang L, Yi H, Chen J, Li H, Luo Y, Cheng T (2022). Neutrophil Extracellular Traps Facilitate A549 Cell Invasion and Migration in a Macrophage-Maintained Inflammatory Microenvironment. Biomed Res Int.

[54] Gao F, Peng H, Gou R, Zhou Y, Ren S, Li F (2025). Exploring neutrophil extracellular traps: mechanisms of immune regulation and future therapeutic potential. Exp Hematol Oncol.

[55] Yu J, Fu Y, Gao J, Zhang Q, Zhang N, Zhang Z (2024). Cathepsin C from extracellular histone-induced M1 alveolar macrophages promotes NETosis during lung ischemia-reperfusion injury. Redox Biol.

[56] Ross EA, Devitt A, Johnson JR (2021). Macrophages: The Good, the Bad, and the Gluttony. Front Immunol.

[57] Carroll MC (2000). The role of complement in B cell activation and tolerance. Adv Immunol.

[58] Mathern DR, Heeger PS (2015). Molecules Great and Small: The Complement System. Clin J Am Soc Nephrol.

[59] Dempsey PW, Allison ME, Akkaraju S, Goodnow CC, Fearon DT (1996). C3d of complement as a molecular adjuvant: bridging innate and acquired immunity. Science.

[60] Lee T, Nair P, Corrigan CJ (2020). Review of monoclonal antibody therapies in asthma and allergic diseases - a new paradigm for precision medicine. Asian Pac J Allergy Immunol.

[61] Gaffen SL, Jain R, Garg AV, Cua DJ (2014). The IL-23-IL-17 immune axis: from mechanisms to therapeutic testing. Nat Rev Immunol.

[62] Smolen JS, Aletaha D, McInnes IB (2016). Rheumatoid arthritis. Lancet.

[63] Brusselle GG, Koppelman GH (2022). Biologic Therapies for Severe Asthma. N Engl J Med.

