# Supplementary material for: Unveiling Interleukin-40: A Novel Regulator of Macrophage and B Cell Function in Allergic Asthma
Source: Int J Biol Sci. 2026 Jun 4;22(11):6115–31. doi: 10.7150/ijbs.128164 (PMC13282775; doi:10.7150/ijbs.128164)
Supplement: Supplementary file 1 — Supplementary figures and tables 1-2. [file ijbsv22p6115s1.pdf]

Supplementary Materials

Unveiling Interleukin-40: A Novel Regulator of Macrophage and B Cell Function in Allergic Asthma

Aixuan Li[1], Katie Ching-Yau Wong[2], Danqi Huang[2,3], Fang Chen[4,5], Haoxuan Li[6], Xun Gao[7,8], Ting-Fan Leung[9], Gary Wing-Kin Wong[9], Wing-Hung Ko[1], Chun-Kwok Wong[2,4,5,10]

[1]School of Biomedical Sciences, The Chinese University of Hong Kong, Hong Kong, China; [2]Department of Chemical Pathology, Prince of Wales Hospital, The Chinese University of Hong Kong, Hong Kong, China; [3]The Eighth Affiliated Hospital of Sun-Yat-sen University, Shenzhen, China; [4]Institute of Chinese Medicine, The Chinese University of Hong Kong, Hong Kong, China; [5]State Key Laboratory of Research on Bioactivities and Clinical Applications of Medicinal Plants, The Chinese University of Hong Kong, Hong Kong, China; [6]Department of Chemistry, Department of Biochemistry and Molecular Biology, and Institute for Biophysical Dynamics, The University of Chicago, Chicago, IL, USA; [7]Center of Clinical Laboratory Medicine, Zhongda Hospital, Southeast University, Nanjing, Jiangsu, China; [8]Department of Laboratory Medicine, Medical School of Southeast University, Nanjing, Jiangsu, China; [9]Departmenmt of Paediatrics, Prince of Wales Hospital, The Chinese University of Hong Kong, Hong Kong, China; [10]Li Dak Sum Yip Yio Chin R & D Centre for Chinese Medicine, The Chinese University of Hong Kong SAR, Hong Kong, China

Correspondence:

Professor Chun-Kwok Wong

Department of Chemical Pathology

The Chinese University of Hong Kong

Prince of Wales Hospital, Shatin, N.T.

Hong Kong, China

Tel: (852) 3505 2964; Fax: (852) 2636 5090; E-Mail: ck-wong@cuhk.edu.hk

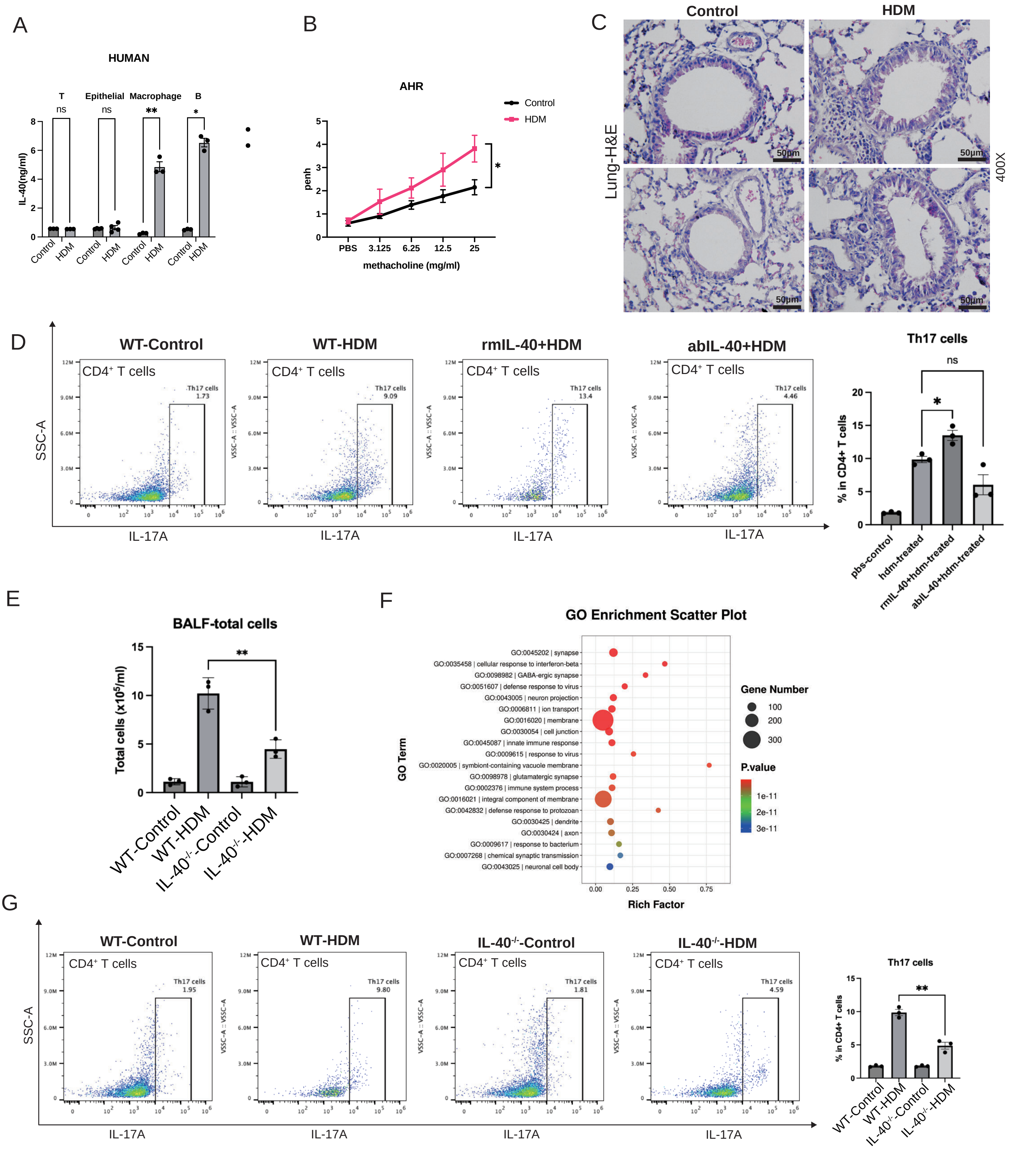

Supplementary Figure 1. IL-40 expression and its effects on airway inflammation and immune cell subsets. **A** Concentrations of IL-40 in sorted human T cells, epithelial cells, macrophages, and B cells measured by ELISA (n=4). **B** AHR to methacholine challenge in control and HDM-treated mice (n=4-5). **C** Representative H&E-stained lung sections from control and HDM-treated groups. **D** Proportion of Th17 cells in lung tissue across groups. **E** Bulk RNA sequencing of lung tissue from WT and IL-40$^{-/-}$ mice, followed by Gene Ontology (GO) analysis of differentially expressed genes (DEGs). **F** Proportion of Th17 cells in lung tissue from WT and IL-40$^{-/-}$ mice. **G** Total cell number in BALF across different groups of mice. Data are representative of at least three independent experiments. Values are expressed as the mean ± SEM. *P < 0.05, **P < 0.01, and ns for not significant by Mann-Whitney test.

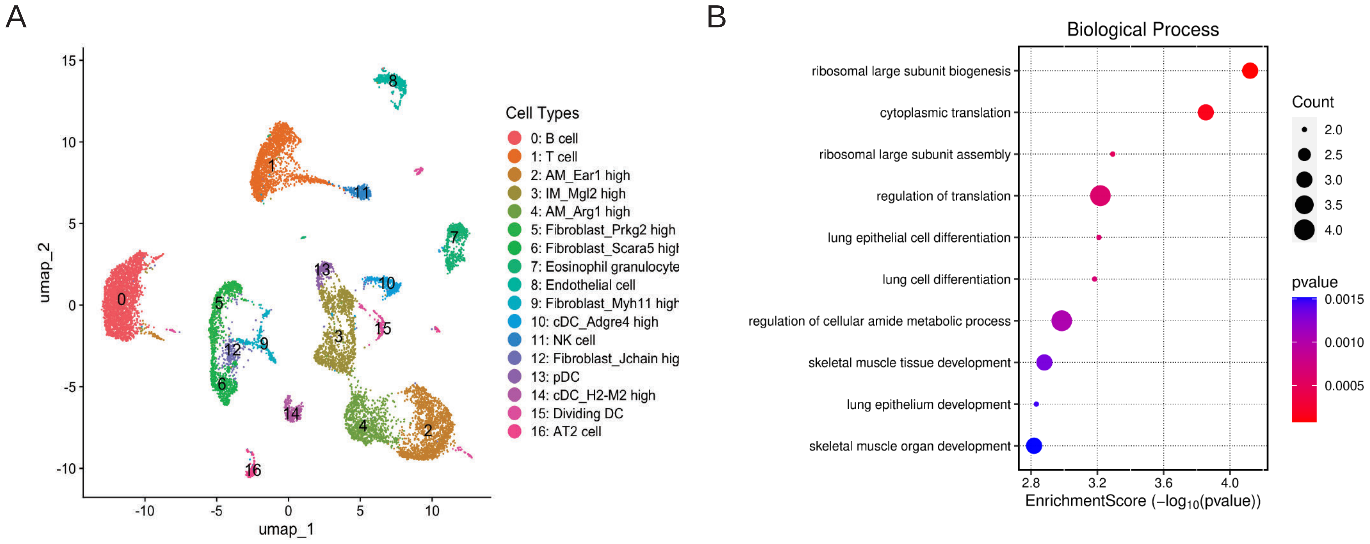

Supplementary Figure 2. IL-40 regulates lung cell clusters and B cell subsets at single-cell resolution. **A** UMAP visualization of annotated pulmonary cell clusters from single-cell RNA sequencing of lung tissue in all mouse groups. **B** Gene ontology (GO) analysis of downregulated differentially expressed genes (DEGs) in cluster 0 (B cell) from IL-40$^{-/-}$ HDM-stimulated mice.

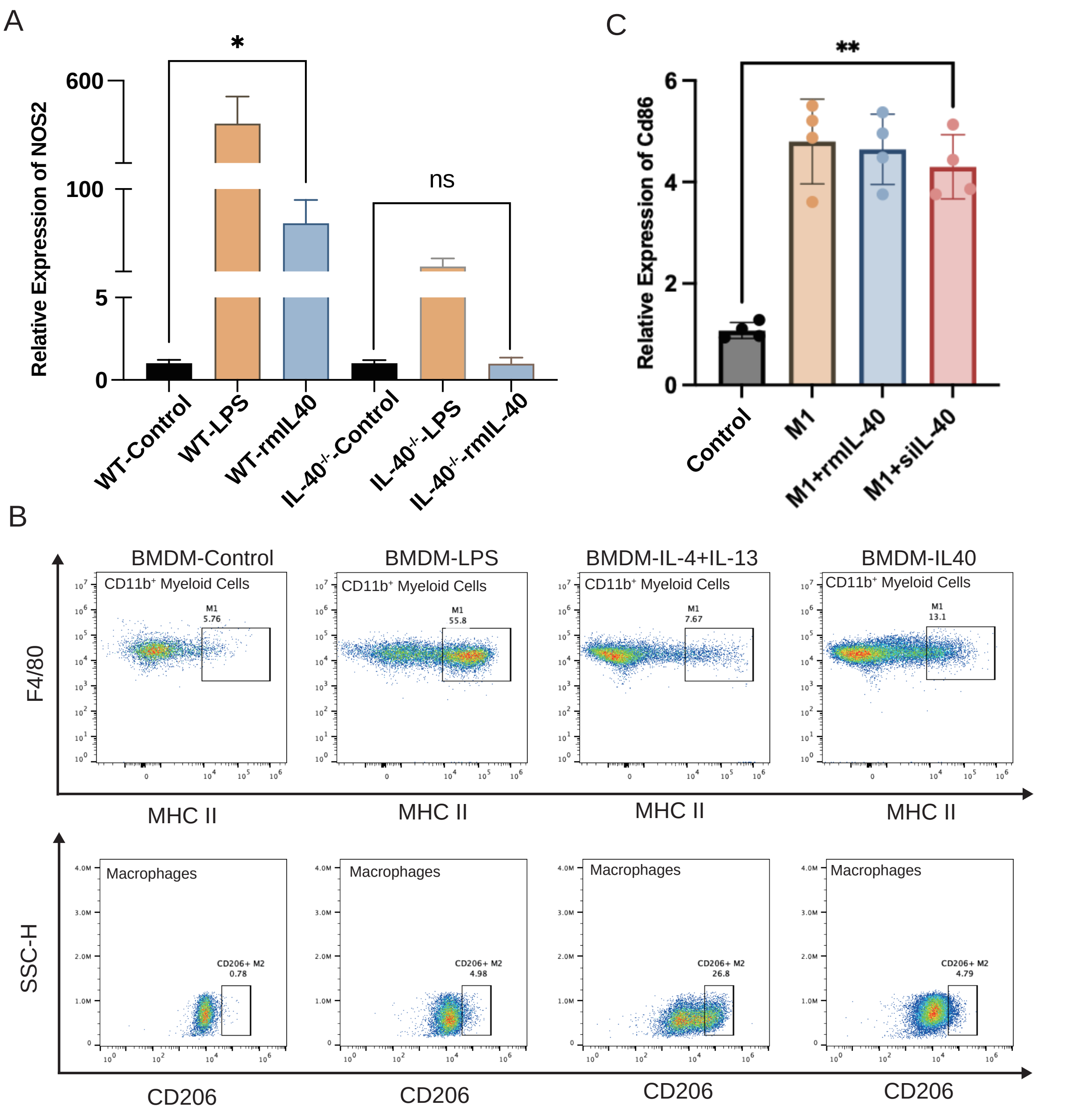

Supplementary Figure 3. IL-40 regulates macrophage polarization. **A, C** Relative expression levels of *NOS2* and *Cd86* in treated BMDMs measured by qRT-PCR (n=4). **B** Flow cytometry analysis of macrophage subtypes following respective stimulations. Values are expressed as mean ± SEM.*P < 0.05; **P < 0.01; ns, not significant by Mann-Whitney test.

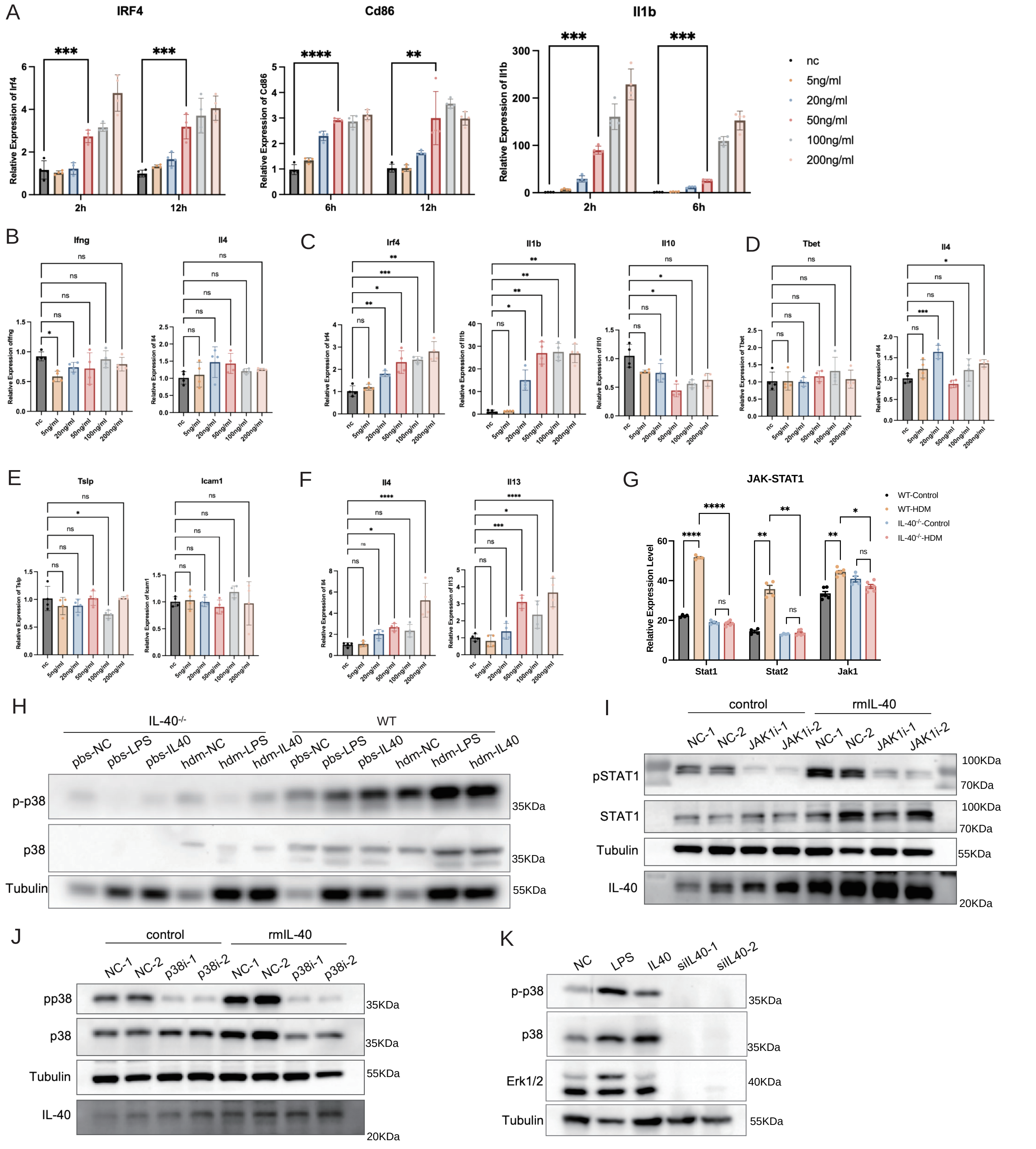

Supplementary Figure 4. IL-40 broadly regulates immune gene and protein expression in multiple cell types. **A** Relative expression levels of *Irf4*, *Cd86* and *Il1b* in treated mouse BMDMs (n=3-4) measured by qRT-PCR. **B** Relative expression levels of *Ifng* and *Il4* in 12h-treated primary T cells isolated from mice thymus (n=3-4) measured by qRT-PCR. **C** Relative expression levels of *Irf4*, *Il1b* and *Il10* in 12h-treated huamn PBMC derived macrophages (n=3-4) measured by qRT-PCR. **D** Relative expression levels of *Tbet* and *Il4* in 12h-treated human T cells isolated from PBMC (n=3-4) measured by qRT-PCR. **E** Relative expression levels of *Tslp* and *Icam1* in 12h-treated HBEpiC (n=4) measured by qRT-PCR. **F** Relative expression levels of *Il4* and *Il13* in 12h-treated mouse T cells from BMDM & T cell coculture sample. **G** Gene expression analysis by bulk RNA-seq in lung tissue from asthmatic and control mice. **H, I, J** Western blot analysis of protein expression in treated mouse BMDMs. **K** Western blot analysis in RAW264.7 cells following indicated treatments. Data are representative of at least three independent experiments. Values are expressed as the mean ± SEM. *P < 0.05; **P < 0.01, ***P < 0.001, ****P < 0.0001 and ns: not significant by the Mann-Whitney test.

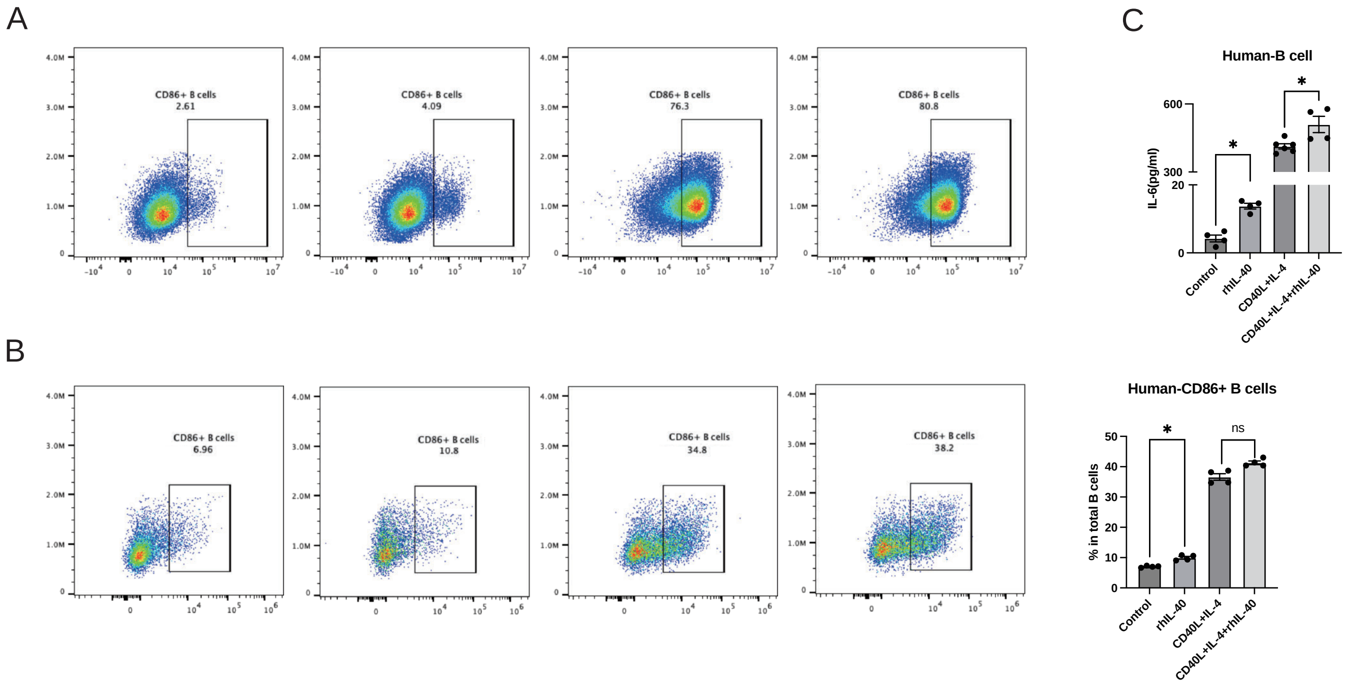

Supplementary Figure 5. IL-40 regulated B cell activation. **A** CD86+ B cells were gated within the CD19$^+$B220$^+$ population in B cells isolated from mouse spleen and **B** human PBMC, representative dot plots and quantification of CD86+ B cells as a percentage of total B cells are shown (n=4-6). **C** Concentrations of IL-6 in cell lysates from human B cells measured by ELISA (n=3-4). Data are representative of at least three independent experiments. Values are expressed as the mean ± SEM. *P < 0.05 and ns: not significant by the Mann-Whitney test.

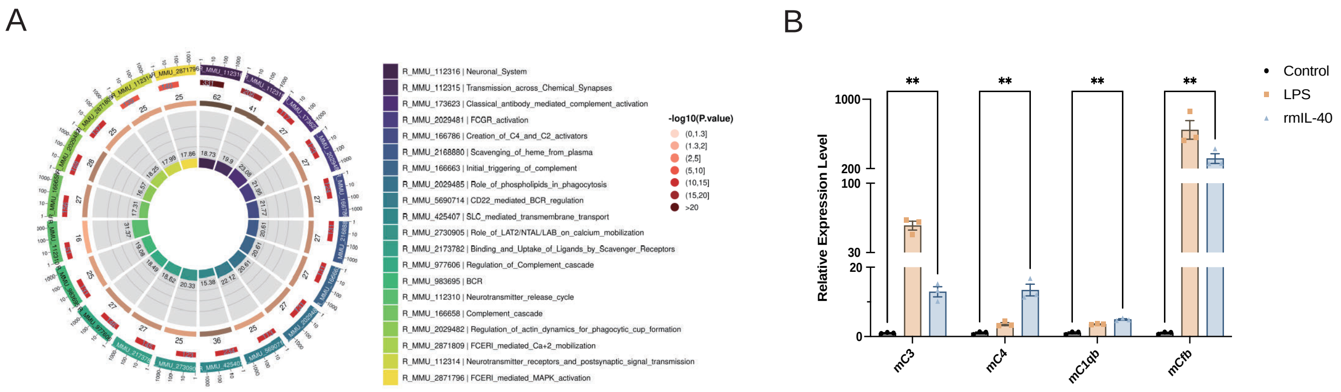

Supplementary Figure 6. IL-40 regulates complement pathway gene expression in macrophages. **A** Circular visualization of Reactome pathway enrichment analysis for complement-related genes. **B** Relative expression levels of complement pathway genes in macrophages measured by qRT-PCR (n=3−5). Data are representative of at least three independent experiments. Values are expressed as the mean ± SEM. **P < 0.01 by the Mann-Whitney test.

Supplementary Table 1. Patient Demographics

Sample ID	Gender	Age	Asthma	AR	Eczema
BC 0065	M	18.0	1	1	0
BC 0532	M	10.5	1	1	0
BC 0076	M	15.1	1	1	0
BC 0630	M	12.1	1	1	0
BC 0481	F	5.2	1	1	0
BC 0051	M	11.4	1	1	0
BC 0069	F	17.1	1	1	0
BC 0446	F	11.8	1	1	0
BC 0085	F	11.8	1	1	0
BC 0048	M	9.2	1	1	0
BC 0056	M	6.8	1	1	0
BC 0426	M	13.3	1	1	0
BC 0500	F	13.7	1	1	0
BC 0474	M	9.8	1	1	0
BC 0853	F	15.3	1	1	0
BC 2282	F	20.1	1	1	0
A 542	M	12.2	1	1	0
A 543	M	6.7	1	1	0
A 545	M	6.4	1	1	0
A 548	M	16.3	1	1	0
A 551	F	7.1	1	1	0
A 555	M	10.9	1	1	0
A 556	F	16.6	1	1	0
A 558	M	9.3	1	1	0
A 562	F	9.4	1	0	0
A 564	F	8.3	1	1	0
A 565	M	8.0	1	1	0
A 567	M	7.7	1	0	0
A 569	M	8.8	1	0	0
A 572	M	12.2	1	1	0
A 575	F	9.1	1	1	0
A 576	F	14.7	1	1	0
A 577	M	10.3	1	1	0
A 580	M	15.3	1	0	0
A 583	F	16.9	1	1	0

A 585	F	8.5	1	1	0
A 586	M	17.4	1	1	0
A 587	M	6.5	1	1	0
A 590	M	8.1	1	1	0
A 597	M	11.5	1	1	0
A 600	M	7.1	1	1	0
A 608	F	12.7	1	1	0
A 609	F	17.3	1	1	0
A 611	M	7.4	1	1	0
A 614	F	7.2	1	0	0
A 615	M	9.7	1	1	0
A 617	F	11.5	1	1	0
A 618	M	16.8	1	1	0
A 620	M	11.0	1	0	0
A 623	M	8.6	1	0	0
A 624	F	7.5	1	0	0
A 632	M	17.9	1	1	0
A 634	M	10.6	1	1	0
A 638	M	13.8	1	1	0
A 641	M	13.5	1	0	0
A 642	M	11.2	1	1	0
A 643	M	10.4	1	1	0
A 645	F	6.2	1	1	0
A 648	M	10.0	1	1	0
A 650	M	11.6	1	1	0
A 656	M	9.9	1	1	0
A 671	F	21.9	1	1	0
A 673	F	9.1	1	1	0
A 678	F	11.2	1	1	0
A 684	M	16.4	1	1	0
A 685	M	10.0	1	1	0
A 687	F	13.4	1	1	0
A 750	F	11.3	1	1	0
A 753	F	9.9	1	1	0
A 758	F	10.1	1	1	0
A 761	M	16.1	1	1	0
A 765	M	9.9	1	0	0
A 770	M	10.0	1	1	0

BC 1585	F	11.4	1	0	0
BC 2760	M	17.5	1	1	0
BC 2798	M	16.0	1	1	0

Supplementary Table 2. Primer sequences for qRT-PCR.

Genes	Primer sequences (5'-3') (Forward)	Primer sequences (5'-3') (Reverse)
Mice Gapdh	TGGAGATGGTGAAAGAGGTG	GTCCGTGGAAATGGTGGC
Mice C3	AAGCATCAACACACCCAACA	CTTGAGCTCCATTCGTGA
Mice C4	AGCGTGTTTCCAGCTCAAAG	GTCCTAAGGCCTCACACCTG
Mice C4b	ACCCCCAGTACTTGCTGGAC	ACCCTGTAGAGCAGAGCCTCTAA
Mice C1qb	TCTGGGAATCCACTGCTGTC	AGACCTCACCCCACTGTGTC
Mice Cfb	CAAGCAGCACAAGGAACAGT	CCTTGGGCCTTTGTAGCATC
Mice Arg1	CATTGGCTTGCGAGACGTAGAC	GCTGAAGGTCTCTTCCATCACC
Mice Cd86	ACGTATTGGAAGGAGATTACAGCT	TCTGTCAGCGTTACTATCCCGC
Mice Nos2	ACTACTGCTGGTGGTGACAA	GAAGGTGTGGTTGAGTTCTCTAAG
Mice Cd206	CTCTGTTCAGCTATTGGACGC	CGGAATTTCTGGGATTCAGCTTC
Mice 6030468B19Rik-1	CATTGCTGACAGGATGCAGAAGG	TGCTGGAAGGTGGACAGTGAGG
Mice 6030468B19Rik-2	GCCTGCAGCTGAGACACTG	GCACCTATGGACCCAGCA
Mice 6030468B19Rik-3	GAATGGTGGGCGTGTTCTTG	GCTCGGGCCTCTGCTC
Human Gapdh	AGCCACATCGCTCAGACAC	GCCCAATACGACCAAATCC
Human C17orf99-1	CAAGGCACGGGAGGAAGAAA	ACAGCAGGTTATGAGCACCC
Human C17orf99-1	CCTGGGCTGTTCTGCTTGG	CGGCCTTTGGGGAAAACTTC
